# Reducing HIV-related stigma and discrimination in healthcare settings: A systematic review of quantitative evidence

**DOI:** 10.1371/journal.pone.0211298

**Published:** 2019-01-25

**Authors:** Garumma Tolu Feyissa, Craig Lockwood, Mirkuzie Woldie, Zachary Munn

**Affiliations:** 1 Jimma University, Department of Health, Behavior and Society, Jimma, Ethiopia; 2 Ethiopian Evidence Based Health Care Centre: JBI Center of Excellence, Jimma University, Jimma, Ethiopia; 3 The Joanna Briggs Institute, the University of Adelaide, Adelaide, Australia; 4 Department of Health Policy and Management, Jimma University, Jimma, Ethiopia; 5 Department of Global Health and Population, T.H. Chan Harvard School of Public Health, Addis Ababa, Ethiopia; Centre for the AIDS Programme of Research in South Africa (CAPRISA), SOUTH AFRICA

## Abstract

**Introduction:**

Stigma and discrimination (SAD) related to HIV compromise access and adherence to treatment and support programs among people living with HIV (PLHIV). The ambitious goal of ending the epidemic of HIV by 2030 set by the United Nations Joint Program of HIV/AIDS (UNAIDS) will thus only be achieved if HIV-related stigma and discrimination are reduced. The objective of this review was to locate, appraise and describe international literature reporting on interventions that addressed HIV-related SAD in healthcare settings.

**Methods:**

The databases searched were: Cumulative Index to Nursing and Allied Health (CINAHL), Excerpta Medica Database from Elsevier (EMBASE), PubMed and Psychological Information (PsycINFO) database. Two individuals independently appraised the quality of the papers using appraisal instruments from the Joanna Briggs Institute (JBI). Data were extracted from papers included in the review using the standardized data extraction tool from JBI. Quality of evidence for major outcomes was assessed using Grading of Recommendations, Assessment, Development and Evaluation (GRADE).

**Results:**

We retained 14 records reporting on eight studies. Five categories of SAD reduction (information-based, skills building, structural, contact-based and biomedical interventions) were identified. Training popular opinion leaders (POLs) resulted in significantly lower mean avoidance intent scores (MD = -1.87 [95% CI -2.05 to -1.69]), mean prejudicial attitude scores (MD = -3.77 [95% CI -5.4 to -2.09]) and significantly higher scores in mean compliance to universal precaution (MD = 1.65 [95% CI 1.41 to 1.89]) when compared to usual care (moderate quality evidence). The Summary of Findings table (SOF) is shown in Table 1.

**Conclusions:**

Evidence of moderate quality indicates that training popular opinion leaders is effective in reducing avoidance intent and prejudicial attitude and improving compliance to universal precaution. Very low quality evidence indicates that professionally-assisted peer group interventions, modular interactive training, participatory self-guided assessment and intervention, contact strategy combined with information giving and empowerment are effective in reducing HIV-related stigma.Further Randomized Controlled Trials (RCTs) are needed. Future trials need to use up-to-date and validated instruments to measure stigma and discrimination.

## Introduction

In the last three decades, the HIV/AIDS epidemic has been one of the most challenging public health problems in the world [1]. Out of the 36.9 million people living with HIV (PLHIV) globally in 2017, only 75% of them knew their HIV positive status. Out of these, only 21.7 million were accessing HIV treatment in 2017 [2]. Currently, there is a global commitment to end the HIV/AIDS epidemic by 2030 [3]. As a roadmap to end the epidemic (as a public health threat) by the year 2030, the United Nations Joint Program of HIV/AIDS (UNAIDS) has set ambitious targets to be achieved by 2020. These targets include making sure 90% of all PLHIV know their sero-status, 90% of those who know their sero-status are receiving treatment, and 90% of PLHIV on treatment having suppressed viral loads [4]. If these targets are to be achieved, the stumbling blocks of stigma and discrimination (SAD) need to be addressed [4, 5].

Although healthcare workers (HCWs) are expected to be a source of comfort, support, and encouragement, it has been documented that they also sometimes stigmatize PLHIV [6, 7]. In healthcare settings, SAD are often manifested in the form of negligence, breaches of confidentiality, gossip, excessive or differential precautions, poor support, delay or denial of treatment, differential treatment, and unnecessary referrals based on the patient’s sero-status [6, 8]. Therefore, some PLHIV are not getting the needed support because of either low healthcare seeking behavior due to fear of stigma, or because of negligence by HCWs [9, 10]. Consequently, HIV-related SAD compromise access to treatment and support programs among PLHIV [10–13]. Stigma and discrimination are also associated with poor physical and mental health outcomes [12, 14, 15], low social support, and reduced income for HIV positive people [15]. As a result, SAD have been contributing to the continued transmission of HIV and its negative impacts [3, 16].

Although there are reviews that addressed interventions targeting HIV-related SAD [17–19], some of them did not report the findings specifically within different population sub-groups (healthcare workers, PLHIV, community members, etc.) and settings (community, healthcare settings, schools, etc.) [17, 18]. The effectiveness of these interventions might have been affected by contextual factors such as the setting (whether the setting is in a healthcare institution or in the community), the policy environment, and other enabling factors present in that setting. These factors were not specified by previous reviews [20, 21]. Though SAD in healthcare settings are presumed to be similar across geographical bounderies, the manifestations of stigma in healthcare settings differs from other contexts, such as schools, community and faith based organizations [7, 22–24].

In healthcare settings, both structural and individual-level factors fuel HIV-related SAD. Individual-level factors that affect the practice of HCWs include fear of casual transmission, and limited knowledge of what stigma is and its negative consequences [7]. Structural factors include shortage of protective equipment, the absence of a redressal system for managing SAD by HCWs, and the asbsence of a supportive policy or presence of a discriminatory policy environment in the healthcare facility [7, 17]. Stigmatizing interactions are also often not recognized by healthcare providers as being stigmatizing [8]. For instance, visibly marking the files of PLHIV is taken as an appropriate practice by some HCWs [8]. The above factors should be addressed through skills building and structural interventions such as availing supplies for standard precautions and establishing non-discrminatory policies and regulations in healthcare facilities [7]. Evidence on the effectiveness and the details of these interventions should be assessed and pooled [21, 25].

Through a preliminary search in PubMed, Cumulative Index to Nursing and Allied Health (CINAHL), Excerpta Medica Database from Elsevier (EMBASE) and Cochrane Library, we found no systematic review addressing stigma and discrimination reduction interventions specific to healthcare settings or HCWs published within the last three years. Therefore, the aim of this review was to identify, appraise and analyze findings of studies reporting on interventions aimed to reduce HIV-related stigma and discrimination among HCWs in healthcare facilities.

### Review questions/objectives

This review sought to locate, appraise and describe international literature reporting on interventions that addressed HIV-related stigma and discrimination among healthcare workers and in healthcare institutions.

Specifically, the review aimed to:

- Identify, appraise and describe studies containing interventions to reduce HIV-related SAD by HCWs.
- Identify, appraise and describe studies that report on institutional-level interventions to reduce HIV-related SAD.

## Methods and participants

This systematic review was prepared using PRISMA reporting guidelines (S1 Table) for systematic reviews [26]. The review was conducted in accordance with the Joanna Briggs Institute methodology for systematic reviews of effectiveness evidence [27] and an *a-priori* protocol registered in PROSPERO 2017 CRD42017071799 (available from http://www.crd.york.ac.uk/PROSPERO/display_record.php?ID=CRD42017071799). During the conduct of the review, we considered the following inclusion criteria.

### Population

This review considered interventions addressing HCWs in healthcare institutions (clinics, health centers, and hospitals). The current review was limited to the context of healthcare institutions; it did not include interventions that were aimed to address SAD in any other locations. We aimed to include not only interventions addressing knowledge gaps of healthcare workers, but also institutional factors contributing to stigma. Some studies that addressed interventions for students, were either a mixture of medical and non-medical students (medical, paramedical and non-health related fields), had only a short-term follow up, or did not address contextual factors within healthcare institutions, such as institutional policies and guidelines, and the presence of materials and supplies [28–30]. Moreover, the level of training of students in the health sciences discipline is not uniform in the studies conducted so far [28–30]. Additionally, HIV services are so sensitive; they most often are handled by permanent staff rather than students, who participate in service only temporarily. Taking these factors into account, we considered only in-service healthcare professionals (those professionals engaged in care provision after graduation) for inclusion.

### Interventions

Interventions that addressed HIV-related SAD by health workers were considered.

These included, but were not limited to the following:

- Information-based approaches including both written and verbal information to increase the understanding of HIV and stigma, provided in the form of leaflets and brochures or through other methods.
- Skills building approach, such as demonstrations and role-plays.
- Structural approaches, such as availing supplies for standard precautions, revision and development of standard operating procedures, polices and regulations, and putting a system in place for addressing grievances.
- Contact strategies, such as testimonials of PLHIV and activities that encourage interaction between HCWs and PLHIV [17, 18].
- Biomedical interventions, such as universal access to care and treatment or expansion of HIV counseling and testing (HCT) [17].
- Counseling and support interventions to help cope with HIV-related stigma and discrimination, specifically secondary stigma (stigma that they may face because of their association with PLHIV) [17]. Studies indicate that HCWs themselves face secondary stigma as a result of their association with PLHIV [31, 32]. It was also shown that HCWs living with HIV faced perceived or actual SAD from colleagues or the community [31].

### Comparators

The comparators considered were baseline (before intervention), no intervention, usual care and one or more of the above components compared to one another.

### Outcomes

The primary outcomes considered for inclusion were HIV-related stigma and discrimination by HCWs in healthcare institutions. Forms of stigma included fear-based stigma, value-based stigma, enacted stigma, internalized stigma, or stigma in other forms. Internalized stigma is defined as the acceptance or internalization of shame, blame, hopelessness, guilt, and fear of discrimination associated with being HIV-positive [33].

HIV-related SAD can be categorized into four domains: drivers, facilitators, manifestations, and intersecting stigma [17]. Drivers are individual level factors that influence the occurrence of stigma. These include lack of adequate knowledge, fear of infection, or prejudicial attitude towards PLHIV or key population groups [17]. Facilitators are organizational or societal level factors that influence stigma. These may include the presence or absence of protective or punitive laws, redress systems, or support systems [17]. Manifestations are the immediate consequences of stigma such as discrimination (experiencing stigma), anticipated stigma, or perceived stigma [34]. Layered or intersecting stigma is the co-occurrence of stigma related to HIV status, gender, profession, poverty or sexual orientation [17]. Studies that used single or separate items and that did not create composite scales of measurement for attitudinal items of stigma were excluded from the review. The secondary outcomes considered were PLHIV-specific extra-precaution (excessive use of precautions or over-use protective barriers selectively when handling PLHIV) and adherence to universal precautions). We considered both dichotomous measures and continuous scale measures for this outcome.

### Context

This review considered all studies conducted worldwide in healthcare settings (hospitals, clinics or health centers).

### Types of studies

This review considered all studies with comparative designs, such as randomized controlled trials (RCTs), quasi-experimental studies and, before and after studies.

### Search strategy

The search strategy aimed to find both published and unpublished studies reported in English. A three-step search strategy was utilized in this review. An initial limited search of Cumulative Index to Nursing and Allied Health (CINAHL) and PubMed was undertaken, followed by an examination of the text words contained in the title and abstract, and of the index terms used to describe the article. A second search using all identified keywords and index terms was then undertaken across all included databases. Thirdly, the reference list of all identified reports and articles was searched for additional studies.

Both published and unpublished papers reported in the English were searched with no restriction to age of the participants, country and date of publication. The databases searched were: CINAHL, Excerpta Medica Database from Elsevier (EMBASE), PubMed and Psychological information (PsycINFO) database. The search for unpublished studies included: HIVinSite, AIDSinfo, HIV and AIDS clearinghouse, Centers for Disease Control and Prevention (CDC) HIV publications, Health Policy Project (HPP) website, United States Agency for International Development (USAID) experience clearinghouse, and United Nations Joint Program on HIV/AIDS (UNAIDS) publications. A detailed search strategy for each database is reported in S2 Table.

### Study selection

Following the above search procedure, all identified citations were loaded into EndNote and duplicates were removed. Titles and abstracts were screened by two independent reviewers for assessment against the inclusion criteria for the review. The full texts of potentially eligible studies were retrieved and assessed in detail against the inclusion criteria by two independent reviewers.

### Assessment of methodological quality

Two individuals (GTF and MS (non-author)) independently appraised the quality of the eligible studies prior to inclusion in the review using appraisal instruments from the Joanna Briggs Institute (JBI) for experimental, quasi-experimental studies, and other comparative study designs [35, 36] (S1 Document). After appraisal, studies that did not meet the methodological criteria were excluded and reasons for their exclusion are provided in S2 Document. All disagreements that arose between the reviewers were resolved through discussion, and there was no requirement for a third reviewer.

### Data extraction and syntheses

Quantitative data were extracted from papers included in the review using the standardized data extraction tool from the JBI (S3 Document) [37]. Relevant information such as population characteristics, publication year, authors, intervention type and summary of the findings were extracted. Where necessary, primary authors were asked to provide additional information on the articles. Details of data from primary studies with limited data or with limited follow up were checked through request to the authors and through checking subsequent publications from the same project, based on cross-checking the linked publications from the registries of trials (if trial registry number existed). For instance, Li et al. 2013 [38] published another article from the same project in 2015 [39].

Effect measures reported in the form of mean difference and standard deviation (for continous variables) and relative risk, odds ratio and their confidence intervals (for dichotomous variables) were extracted and reported. Since the studies were methodologically or clinically heterogeneous, statistical pooling was not possible; hence, the findings are presented in narrative form. The quality of evidence for major outcomes reported in each study was assessed using a software package developed by the Grading of Recommendations, Assessment, Development and Evaluation (GRADE) [40] working group.

## Results

**Fig 1:**
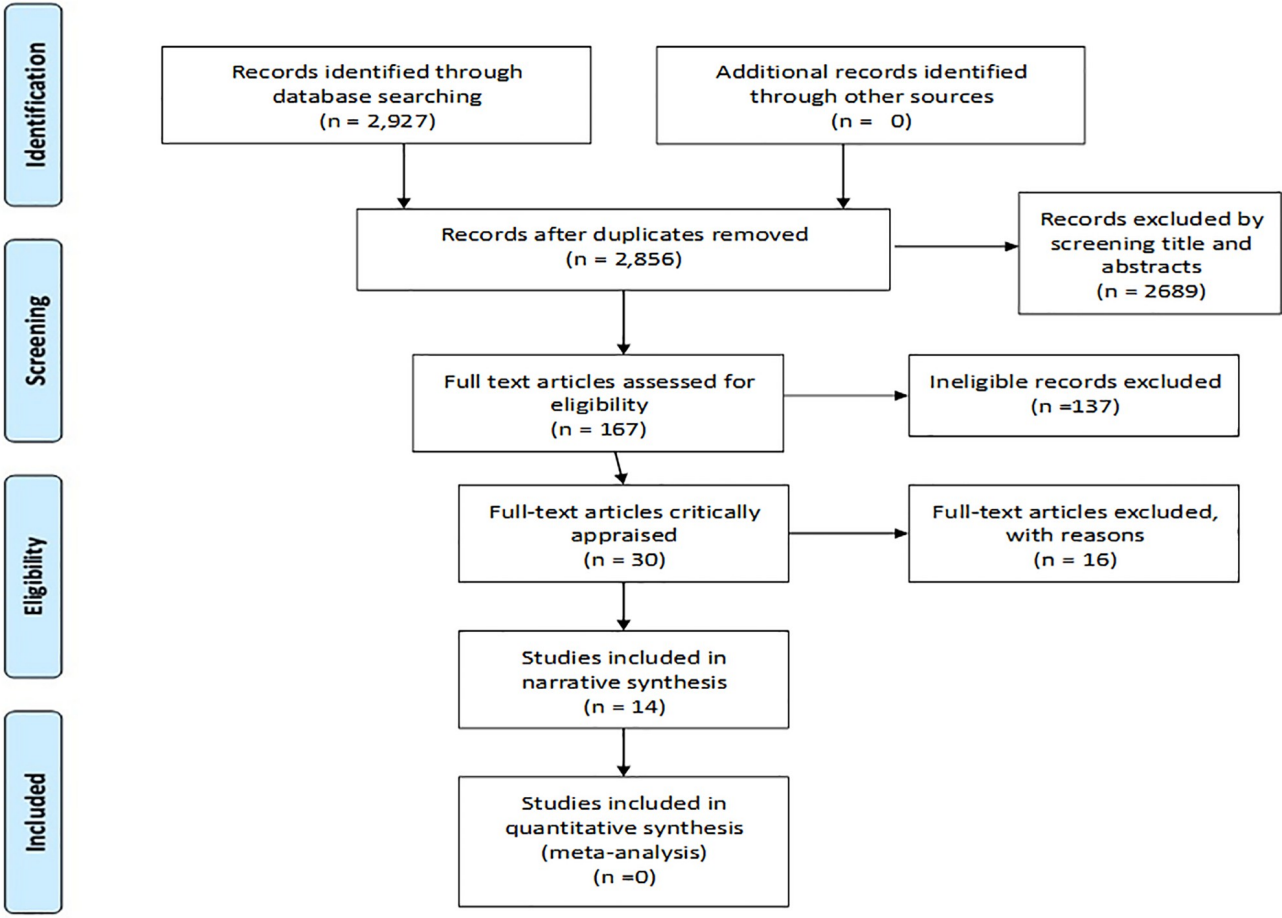
Study selection process. *From*: Moher D, Liberati A, Tetzlaff J, Altman DG, The PRISMA Group (2009). *P*referred *R*eporting *I*tems for *S*ystematic Reviews and *M*eta-*A*nalyses: The PRISMA Statement. PLoS Med 6(7): e1000097. doi:10.1371/journal.pmed1000097.

The search yielded a total of 2,927 records. After removing, duplicates, 2,856 documents were retained for further examination. After screening the titles and abstracts, 167 records were retained for full text examination. Based on pre-defined inclusion criteria, 30 records were included for critical appraisal. Finally, 14 records reporting on eight studies were retained (Fig 1). Sixteen studies [41–56] were excluded based on reason. Almost all studies excluded based on reason had significant measurement bias.

### Description of the characteristics of included studies

Among the 14 records included in this review, six articles (Li et al. 2013a [57]; Li et al. 2013b [58]; Li et al. 2013c [38]; Li et al. 2014a [59]; Li et al. 2014b [60]; and Li et al. 2015 [39]) reported on the findings of a single randomized trial. The trial was conducted in 40 hospitals of China. Hereafter, Li et al. 2015 [39] will be used to describe the findings extracted from the study, although data were extracted from all the six articles to get complete information. In addition, another before-and-after study (Williams et al. 2006 [61]) was conducted in China. The other studies were conducted in Chile (Norr et al. 2012 [62]); India (Mahendra et al. 2006 [63]);Vietnam (Pulewitz et al. 2015 [64] and Oahn et al. 2008 [65]); Egypt (Lohiniva et al. 2016 [66]); USA (Zachary 1998 [67]) and one study was a multi-country case study (Uys et al. 2009 [68]). Pulewitz et al. 2015 [64] and Oahn et al. 2008 [65] both reported on the same study. For reporting purpose, hereafter, we will use Pulewitz et al. 2015 [64]. The characteristics of the included studies are shown in Table 1.

**Table 1:** Study characteristics.

Study ID	Study location	Category of intervention	Type of intervention versus comparison	Participants	Follow up duration	Level of implementation	Domain of stigma	Outcomes
Li et al. 2015 [39]	China	Information-based, skills building and structural	Identifying and training POL through group discussion, games, and role-plays versus usual care (supplies provided for both arms)	1740 (880 control and 880 intervention) HCWs from 40 hospitals	12 months	Organizational, individual	Driver, facilitator	Prejudicial attitude, avoidance intent, adherence to universal precaution, and institutional support
Lohiniva et al. 2016 [66]	Egypt	Information-based, skills building and contact	Interactive training and discussion focusing on HIV-related stigma, infection control and medical ethics combined with contact with PLHIV (5 modules)	347 (203 intervention and 144 control) HCWs from 2 hospitals	4 months	Individual	Driver	Value-based stigma, fear-based stigma
Norr et al. 2012 [62]	Chile	Information-based	8 sessions of professionally-assisted peer group intervention	555 (293 control and 262 intervention) HCWs from 5 clinics	3 months	Individual	Driver	Public contact stigma, client contact stigma, blame
Mahendra et al. 2006 [63]	India	Information-based, skills building, structural, contact and biomedical approach	Participatory self-guided assessment and intervention with training, development and dissemination of guidelines and educational materials on infection control	884 HCWs in pre-test and 885 HCWs post-test from 3 hospitals	6-months	Organizational, individual	Driver, facilitator, manifestation	Stigmatizing beliefs and practices
Pulewitz et al. 2015 [64]	Vietnam	Information-based, skills building, structural and contact	Arm 1: 1-day workshop and 1.5-day training on HIV/AIDS basic knowledge and universal precaution Arm 2: SAD in addition to interventions in Arm 1	795 HCWs at baseline and 797 HCWs at end line	6 months	Organizational, individual	Driver, facilitator, manifestation	Fear-based stigma, social stigma, enacted stigma
Williams et al. 2006 [61]	China	Information-based, skills building	A 5-day workshop comprising didactic lectures	180 nurses at baseline and 180 nurses post intervention	5 days	Individual	Driver	AIDS attitude, willingness to carry out nursing activities for PLHIV
Uys et al. 2009 [68]	Lesotho, Malawi, South Africa, Swaziland, and Tanzania	Information-based, skills building, and contact	A 2-day workshop bringing PLHIV and nurses together	43 nurses and 41 PLHIV	1 month	Organizational, individual	Driver, manifestation	stigma, self-efficacy and self-esteem
Zachary 1998 [67]	USA	Information-based	A 1-hour group education on homophobia and fear of death	35 nurses in one medical Centre	Only post intervention data (No follow up)	Individual	Driver	AIDS phobia, homophobia

NB: POL: Popular opinion leaders, HCWs: Healthcare workers, SAD: Stigma and discrimination,HIV: Human immunodeficiency virus, PLHIV: People Living with HIV, USA: United States of America, AIDS: Acquired immunodeficiency syndrome.

### Methodological quality of individual studies

Six articles (Li et al. 2013a [57]; Li et al. 2013b [58]; Li et al. 2013c [38]; Li et al 2014a [59]; Li et al. 2014b [60] and Li et al. 2015 [39]) reported on the findings of a single randomized controlled trial. None of the included articles clearly described the method of randomization and allocation concealment. Three studies (Norr et al. 2012 [62]; Lohiniva et al. 2016 [66] and Pulewitz et al. 2015 [64]) were non-randomized trials with control groups. Two before and after trials without control groups (Williams et al. 2006 [61] and Zachary 1998 [67]) had post intervention data taken from the same cohort of participants as those of baseline participants. One study (Uys et al. 2009 [68]) was a multiple-case study design reporting on the same cohort of nurses and PLHIV before and after the intervention. One before and after study without a control group (Mahendra et al. 2006 [63]) reported on independent cross-sectional samples from the same institution. The study did not identify and control for confounding factors.

Participant blinding is not practical for such behavioral interventions. Hence, none of the studies described blinding of participants or assessors, and outcome concealment. Five of the studies (Li et al 2015 [39]; Lohiniva et al. 2015 [66]; Pulewitz et al. 2015 [64]; Norr et al. 2012 [62] and Zachariah 1998 [67]) used self-administered questionnaires. Five studies (Lohiniva et al 2016 [66]; Williams et al. 2006 [61]; Pulewitz et al. 2015 [64]; Mahendra et al. 2006 [63] and Uys et al. 2009 [68]) were judged to have unclear or a high risk of bias on outcome assessment, because either there was significant loss to follow up or inadequate information (Lohiniva et al. 2016 [66] and Williams et al. 2006 [61]), or they used repeated cross-sectional samples with different samples (Mahendra et al. 2006 [63]), or there was a considerable proportion of participants with incomplete data who were excluded from analyses (Pulewitz et al. 2015 [64]). Overall, the included RCT (Li et al. 2015 [39]) scored 8/13 using the scoring method for measuring the methodological qualitieas of RCTs (Table 2). One of the quasi-experimental studies (Norr et al. 2012 [62]) scored 9/13. Four quasi-experimental studies studies ((Lohiniva et al. 2016 [66]; Williams et al. 2006 [61] and Pulewitz et al. 2015 [64]) scored 8/9 (Table 3). One repeated cross-sectional study (Mahendra et al. 2006 [63]) scored 6/8 (Table 4). The multiple case study (Uys et al. 2009 [68]) was given a score of 8/10 (Table 5).

**Table 2:** Methodological quality of randomized controlled trails.

Study ID	Q1	Q2	Q3	Q4	Q5	Q6	Q7	Q8	Q9	Q10	Q11	Q12	Q13	Total of ‘yes’ scores
Li et al. 2015 [39]	U	U	Y	U	U	U	Y	Y	Y	Y	Y	Y	Y	8

**NB**: Y = Yes, U = unclear, NA = not applicable

Q1. Was true randomization used for assignment of participants to treatment groups?

Q2. Was allocation to treatment groups concealed?

Q3. Were treatment groups similar at the baseline?

Q4. Were participants blind to treatment assignment?

Q5. Were those delivering treatment blind to treatment assignment?

Q6. Were outcomes assessors blind to treatment assignment?

Q7. Were treatments groups treated identically other than the intervention of interest?

Q8. Was follow-up complete, and if not, were strategies to address incomplete follow-up utilized?

Q9. Were participants analyzed in the groups to which they were randomized?

Q10. Were outcomes measured in the same way for treatment groups?

Q11. Were outcomes measured in a reliable way?

Q12. Was appropriate statistical analysis used?

Q13. Was the trial design appropriate, and any deviations from the standard RCT design (individual randomization, parallel groups) accounted for in the conduct and analysis of the trial?

**Table 3:** Methodological quality of quasi-experimental studies.

S/N	Study ID	Q1	Q2	Q3	Q4	Q5	Q6	Q7	Q8	Q9	Total of ‘yes’ scores
1.	Lohinva et al. 2015 [66]	Y	Y	Y	Y	Y	N	Y	Y	Y	8
2.	Norr et al. 2012 [62]	Y	Y	Y	Y	Y	Y	Y	Y	Y	9
3.	Pulewitz et al. 2015 [64]	Y	Y	Y	Y	Y	N	Y	Y	Y	8
4.	Williams et al. 2006 [61]	Y	Y	Y	N	Y	U	Y	Y	Y	8
5.	Zachariah 1998 [67]	Y	Y	Y	N	Y	Y	Y	Y	Y	8

**NB**: Y = Yes, U = unclear, NA = not applicable

Q1. Is it clear in the study what is the ‘cause’ and what is the ‘effect’ (i.e. there is no confusion about which variable comes first)?

Q2. Were the participants included in any comparisons similar?

Q3. Were the participants included in any comparisons receiving similar treatment/care, other than the exposure or intervention of interest?

Q4. Was there a control group?

Q5. Were there multiple measurements of the outcome both pre-and post the intervention/exposure?

Q6. Was follow up complete and if not, were differences between groups in terms of their follow up adequately described and analyzed?

Q7. Were the outcomes of participants included in any comparisons measured in the same way?

Q8. Were outcomes measured in a reliable way?

Q9. Was appropriate statistical analysis used?

**Table 4:** Summary score for methodological quality of repeated cross-sectional studies.

Study ID	Q1	Q2	Q3	Q4	Q5	Q6	Q7	Q8	Total of ‘yes’ scores
Mahendra et al. 2006 [63]	Y	Y	Y	Y	N	N	Y	Y	6

**NB:** Y = Yes, U = unclear, NA = not applicable

Q1. Were the criteria for inclusion in the sample clearly defined?

Q2. Were the study subjects and the setting described in detail?

Q3. Was the exposure measured in a valid and reliable way?

Q4. Were objective, standard criteria used for measurement of the condition?

Q5. Were confounding factors identified?

Q6. Were strategies to deal with confounding factors stated?

Q7. Were the outcomes measured in a valid and reliable way?

Q8. Was appropriate statistical analysis used?

**Table 5:** Summary score for methodological quality of case series studies.

Study ID	Q1	Q2	Q3	Q4	Q5	Q6	Q7	Q8	Q9	Q10	Total of ‘yes’ scores
Uys et al 2009 [68]	Y	Y	Y	NA	Y	Y	NA	Y	Y	Y	8

**NB:** Y = Yes, U = unclear, NA = not applicable

Q1. Were there clear criteria for inclusion in the case series?

Q2. Was the condition measured in a standard, reliable way for all participants included in the case series?

Q3. Were valid methods used for identification of the condition for all participants included in the case series?

Q4. Did the case series have consecutive inclusion of participants?

Q5. Did the case series have complete inclusion of participants?

Q6. Was there clear reporting of the demographics of the participants in the study?

Q7. Was there clear reporting of clinical information of the participants?

Q8. Were the outcomes or follow up results of cases clearly reported?

Q9. Was there clear reporting of the presenting site(s)/clinic(s) demographic information?

Q10. Was statistical analysis appropriate?

### Findings of the studies

Interventions used in studies included in this review fell under five general categories: information-based interventions, structural interventions, biomedical interventions, and skills building, and contact strategies. Most studies utilized more than one of the above general categories of interventions. One study (Mahendra et al 2006 [63]) used all of the five categories of interventions in combination. One study (Pulewitz et al. 2015 [64]) utilized a combination of information-based, skills building, structural and contact approaches. Two studies (Uys et al. 2009 [68] and Lohiniva et al. 2016 [66]) used a combination of information-based, skills building, and contact approaches. One study (Li et al. 2015 [39]) utilized a combination of information-based, skills building and structural approaches. One study (Williams et al. 2006 [61]) utilized a combination of information-based and skills building approaches. The remaining two studies (Norr et al. 2012 [62] and Zachary 1998 [67]) utilized only information-based intervention.

Since the design, duration of follow-up, and instruments used to measure the effect of the interventions were not uniform across the included studies, we could not compare one type of intervention with another, nor multiple interventions with a single intervention. We also could not pool the findings of the studies using meta-analysis. Nevertheless, we present the findings of the studies along with their respective quality of evidence. The details of the intervention and their effects are described in detail below.

#### Training popular opinion leaders

The study by Li et al.[39] identified and trained popular opinion leaders (POLs) through group discussions, games, and role-plays. The POLs attended a 1.5-hour weekly group training session run for four weeks and fortnightly re-union sessions. Materials for universal precaution were supplied in both control and intervention hospitals. The authors collected data from 880 HCWs from 20 intervention hospitals (where POLs were trained) and from another 880 HCWs from 20 control hospitals.

HCWs’ avoidance intent was measured using a five-point Likert-scale which assessed HCWs’ willingness to treat PLHIV in eight scenarios. Higher scores indicated a higher intent to avoid service provision to PLHIV. The intervention effect on avoidance intent was sustained even at 12 months follow up. At 12 months follow up, avoidance intent among HCWs in the intervention hospitals was significantly lower (mean difference (MD) = -1.87 [95% CI -2.05 to -1.69]) when compared to that of healthcare workers in control hospitals (P<0.01). Prejudicial attitude was measured using eight items rated on the five-point Likert scale, however, only effect estimate was reported. At 12 months follow up, the prejudicial attitude among healthcare workers in the intervention hospitals was significantly lower (MD = –3.77 [95% CI -5.4 to -2.09]) when compared to that of HCWs in control hospitals (P<0.01). Universal precaution (UP) compliance was measured using 13 items with responses ranging from 0 (never) to 4 (always) on a Likert scale. Higher scores indicate higher levels of adherence to UP. Universal precaution compliance was significantly higher among HCWs in the intervention group (MD = 1.65 [95% CI 1.41 to 1.89]) when compared to HCWs assigned to usual care (P<0.01) [moderate quality of evidence].

#### Modular interactive training and discussion

The study by Lohiniva et al.[66] assessed the effect of an interactive training and discussion intervention focusing on HIV-related stigma, infection control, and medical ethics using five modules. The intervention was complemented by interaction with PLHIV. The authors assessed the effect of the training by collecting pre-intervention and post-intervention data from the same cohort of participants. They used nine items to measure value-based stigma. Twelve items were used to measure fear-based stigma. Both scales were standardized to obtain scores ranging from 1 to 10. For both scales higher scores indicated higher levels of stigma. At the post-intervention survey, the participants from the experimental group reported significantly lower levels of value-based stigma (mean = 2.1) when compared to participants in the control group (mean = 3.8) (MD = -1.7, P<0.01, CI was not given). Similarly, fear-based stigma was significantly lower among participants in the experimental group (mean = 1.1) when compared to that of participants in the control group (mean = 3.2) (MD = -2.1, P<0.01, CI was not given) [very low quality evidence].

#### Professionally-assisted peer group intervention

The study by Norr et al. [62] assessed eight sessions of professionally-assisted peer group interventions. The sessions covered (a) the importance of community HIV prevention; (b) standard precautions in the healthcare setting; (c) HIV testing and treatment; (d) offering care that respects human dignity and confidentiality; (e) human sexuality, sexual transmission of HIV and other sexually transmitted infections (STIs), and HIV transmission through drug use and blood; (f) partner communication and HIV prevention;(g) counselling about HIV infection; and (h) HIV prevention to clients and families using role plays.

To assess the effect of the intervention, pre-and post-intervention measurements were made for both control and intervention groups. Public contact stigma was measured using three items on a four-point Likert scale. Since mean item scores (instead of the mean scale score) were reported, possible scores ranged from 1 to 4. Client contact stigma was measured using three items on a three-point Likert scale. Again, mean item scores were reported, so possible scores ranged from 1 to 3. For both scales, higher scores indicated a higher level of stigma. After the intervention, the level of client contact stigma among participants in the intervention group was significantly lower [MD = -0.28 (95% CI -0.37 to -0.19)] (P<0.01) [very low quality of evidence]. Similarly, public contact stigma among the intervention group was significantly lower (MD = -0.07 (95% CI -0.12 to -0.02) (P<0.01) [very low quality of evidence].

#### Staff training, participatory hospital policy development, provision of materials and supplies, and expansion of HCT services

Under this group of interventions, there are two subtypes of interventions reported in the studies included in this review:

**a. Participatory self-guided assessment and intervention with training and the development and dissemination of policy guidelines and educational materials.** The study by Mahendra et al.[63] conducted a participatory self-guided assessment and intervention with interactive training facilitated by representatives of AIDS service organizations (including PLHIV), which involved the development and dissemination of policy guidelines and educational materials, such as posters on infection control and expansion and strengthening of HIV counseling and testing (HCT) in three hospitals. The effect of the intervention was measured using an index score of stigma that could range from a minimum of 21 to a maximum of 63, a higher score indicating greater level of stigma. They assessed the effect of the intervention using repeated cross-sectional surveys. After the intervention, there was a significant decrease in stigma score from 42.79 at baseline to 38.08 after the intervention (MD = -4.72, CI not given, P<0.01) [very low quality evidence].

In addition, the probability of seeking informed consent to test for HIV was 2.14 (95% CI 1.17 to 3.91) times higher after the intervention when compared to the likelihood before the intervention (P<0.01) [very low quality evidence]. The likelihood of using gloves after the intervention was 7.81 (95% CI 3.64 to 16.76) times higher after the intervention when compared to the likelihood before the intervention (P<0.01) [very low quality evidence].

**b. Staff training, participatory hospital policy development and provision of material supplies.** The study by Pulewitz et al.[64] compared the effect of addressing fear-based stigma alone (arm 1) to that of addressing both fear-based stigma and social stigma (stemming from moral judgments) (arm 2) through interventions that encompassed staff training, participatory hospital policy development and provision of materials and supplies. Healthcare workers in arm 1 received interventions that addressed only fear-based stigma. The interventions included a half-day training on basic knowledge of HIV/AIDS and a one-day training on universal precaution. Healthcare workers in arm 2 received interventions that addressed both fear-based stigma and social stigma. The training for arm 2 participants encompassed basic knowledge of HIV, universal precaution and stigma. The authors reported that both interventions had significantly reduced stigma.

The authors measured fear-based stigma using four items with alternative responses ranging from 1 (no fear) to 3 (a lot of fear). Hence, the composite score ranged from 4 to 12. Social stigma was measured using five items, each having a score range of 1 to 3. The composite score ranged from 5 to 15. In both scales, higher scores indicated a higher level of stigma.

After the intervention, HCWs exposed to interventions that addressed both fear-based stigma and social stigma had significantly lower scores (MD = -0.37, 95% CI -0.54 to -0.21) of fear-based stigma when compared to those HCWs exposed to interventions that addressed only fear-based stigma (P<0.01) [Very low quality evidence]. On the other hand, there was no statistically significant difference in social stigma (MD = -0.14, 95% CI -0.43 to 0.15) between the two intervention groups [very low quality evidence].

The odds of over-using protective barriers was 46% (OR 0.54 (95% CI 0.31 to 0.91) lower among HCWs exposed to interventions addressing both fear-based and social stigma when compared to those of HCWs exposed to intervention that addressed fear-based stigma alone (P<0.01) [Very low quality evidence]. The odds of marking files of HIV positive clients was 46% (OR 0.54 (95% CI 0.29 to 1) lower among HCWs exposed to interventions addressing both fear-based and social stigma when compared to those of HCWs exposed to interventions that addressed fear-based stigma alone (P<0.01) [very low quality evidence]. The odds of putting signs on a patients bed indicating HIV status was 75% (OR 0.25 (95% CI 0.07 to 0.87)) lower among HCWs exposed to interventions addressing both fear-based and social stigma when compared to those of HCWs exposed to interventions that addressed fear-based stigma alone (P<0.01) [very low quality evidence].

#### Multifaceted educational programs comprising didactic lectures and activities eliciting discussions

The study by Williams et al.[61] investigated a five-day workshop for HCWs comprising didactic lectures on HIV/AIDS epidemiology, natural history, transmission routes and clinical care combined with activities that provoked discussion of participants’ values and personal feelings about HIV/AIDS. To measure the effect of the intervention, both empathy and avoidance attitude scores ranged from 1 to 6. Higher scores in empathy and lower scores in avoidance attitude indicated a more desirable attitude. General attitude score was calculated by substracting the avoidance score from the empathy score. It ranged from -5 to 5. Positive general attitude scores suggested a supportive attitude, while negative scores suggested a negative attitude. The nurses’ willingness questionnaire (NWQ) was used to measure the willingness of nurses to perform activities on HIV-positive patients. The questionnaire was composed of 13 items measured on an 11-point Likert scale ranging from 0 (not all willing) to 10 (extremely willing). Hence the total possible scores ranged from 0 to 130.

The intervention resulted in significant improvement in empathy (MD = 0.2 (CI not given, P<0.01); reduction in avoidance attitude (MD = -0.4, CI not given, P<0.01), improvement in general attitude towards PLHIV (MD = 0.6, CI not given, P<0.01), and willingness to care for PLHIV (MD = 13, CI not given, P<0.01) [Very low quality of evidence].

#### Contact strategy: Workshops bringing PLHIV and Healthcare workers together

The multi-country case study by Uys et al.[68] assessed the impact of a two-day workshop that brought PLHIV and nurses together. The authors measured self-esteem using Rosenberg’s self-esteem scale, which consists of ten items rated on a four-point Likert scale. Scores could range from 10 to 40. Stigma experienced by PLHIV was measured using 33 items. It had six domains: verbal abuse, negative self-perception, healthcare neglect, social isolation, fear of contagion and workplace stigma. An HIV/AIDS stigma instrument that contained 19 items was used to measure stigmatizing attitudes among nurses. The authors reported stigma items in mean scores, but did not indicate the possible ranges for mean scores.

After the intervention, there was a significant increase in self-esteem (MD = 2.12 (95% CI 0.18 to 4.06)) (P<0.05), while there was a decrease in experience of workplace stigma [MD = -0.31 (95% CI -0.61 to -0.01)) (P<0.05), total stigma score (MD = -0.17 (95% CI -0.35 to -0.01)) (P<0.01), and negative self-perception (MD = -0.46 (95% CI -0.81 to -0.11)) (P<0.01) among PLHIV [Very low quality evidence]. On the other hand, there was no significant effect observed on PLHIV’s experience of verbal abuse (P = 0.38), healthcare neglect (P = 0.24), social isolation (P = 0.51) and fear of contagion (P = 0.29). Similarly, there was no significant change in nurses’ stigmatizing attitudes after the intervention (MD = 0.07 (95% CI -0.04 to 0.18)) (P = 0.37) [very low quality evidence].

#### Group education on homophobia and fear of death

The study by Zachary [67] used group education on AIDS phobia, homophobia and fear of death. The study measured AIDS phobia using 15 items rated on a five-point Likert scale. Hence, the score could range from 15 to 75. The intervention did not result in a significant reduction in AIDS phobia (MD = 0.03 (-3.13 to 3.19) (P = 0.94) [Very low quality evidence].

The Summary of Findings (SoF) for all outcomes extracted from all studies are presented in a single table (Table 6).

**Table 6:** Summary of Findings.

Outcomes	Illustrative comparative risks (95% CI)		Relative effect (95% CI)	No of Participants (studies)	Quality of the evidence (GRADE)
	Assumed risk	Corresponding risk			
	**Control**	**Training popular opinion leaders**			
**Avoidance intent** Scale from: 8 to 40. Follow-up: mean 12 months	The mean avoidance intent in the control groups was 18.65	The mean avoidance intent in the intervention groups was 1.87 lower (2.05 to 1.69 lower)		1760 (1 study)	⊕⊕⊕⊝ moderate^1^
**Prejudicial attitude** Scale from 8 to 40 Follow-up: mean 12 months	The mean prejudicial attitude in the control groups was not given	The mean prejudicial attitude in the intervention groups was 3.77 lower (5.4 to 2.09 lower)		1760 (1 study)	⊕⊕⊕⊝ moderate^2^
**Universal precaution compliance** Scale from: 0 to 52. Follow-up: mean 12 months	The mean UP compliance in the control groups was 32.88	The mean UP compliance in the intervention groups was 1.65 higher (1.42 to 1.89 higher)		1760 (1 study)	⊕⊕⊕⊝ moderate^1,2^
	**Control**	**Peer education of HCWs**			
**Public Contact stigma** Scale from: 1 to 3	The mean public contact stigma in the control groups was 1.11	The mean blame in the intervention groups was 0.07 lower (0.12 to 0.02 lower)		927 (1 study)	⊕⊝⊝⊝ very low^3^
**Client contact stigma** Scale from: 1 to 4 Follow-up: mean 12 months	The mean client contact stigma in the control groups was 1.81	The mean contact stigma in the intervention groups was 0.28 lower (0.37 lower to 0.19 lower)		927 (1 study)	⊕⊝⊝⊝ very low^3,4^
	**Control**	**Interactive training and discussion focusing on HIV-related stigma, infection control and medical ethics and contact with PLHIV**			
**Fear-based stigma** Scale from: 1 to 10 Follow-up: mean 3 months	The mean fear-based stigma in the control groups was 3.2	The mean fear-based stigma in the intervention groups was 2.1 lower (CI not given, P<0.01)		347 (1 study)	⊕⊝⊝⊝ very low^5,6^
**Value-based stigma** Scale from: 1 to 10 Follow-up: mean 3 months	The mean value-based stigma in the control groups was 3.8	The mean value-based stigma in the intervention groups was 1.7 lower (CI not given, P<0.01)		347 (1 study)	⊕⊝⊝⊝ very low^5^
	**Control**	**Participatory self-guided assessment and intervention**			
**Stigma index** Scale from 21 to 63 Follow-up: mean 6 months	The mean stigma index (attitude towards PLHIV and healthcare-related practices) in the control groups was 42.79	The mean stigma index (attitude towards PLHIV and healthcare related practices) in the intervention groups was 4.72 lower (CI not given, p<0.01)		1769 (1 study)	⊕⊝⊝⊝ very low^7,8^
**Use of glove when drawing blood if sero-status is unknown** Follow-up: mean 6 months	Study population		RR 7.81 (3.64 to 16.76)	269 (1 study)	⊕⊝⊝⊝ very low^7,8^
	642 per 1000	1000 per 1000 (1000 to 1000)			
**Sought informed consent before HIV test** Follow-up: mean 6 months	Study population		RR 2.14 (1.17 to 3.91)	177 (1 study)	⊕⊝⊝⊝ very low^7,8^
	403 per 1000	862 per 1000 (471 to 1000)			
	Addressing both fear-based and social stigma (stemming from moral judgments).	Addressing ‘fear-based’ stigma (stemming from lack of knowledge)			
	The mean fear-based stigma in the control groups was 5.1	The rate of change in mean fear-based stigma in the intervention groups was 0.37 lower (0.54 to 0.21 lower)		797 (1 study)	⊕⊝⊝⊝ very low^9^
**Social stigma** Scale from 5 to 15 Follow-up: mean 6 months	The mean social stigma in the control groups was 7.4	The rate of change in mean social stigma in the intervention groups was 0.14 lower (0.43 lower to 0.15 higher)		797 (1 study)	⊕⊝⊝⊝ very low^9^
**Overusing any form of barrier protection** Follow-up: mean 6 months	Study population		OR 0.54 (0.31 to 0.91)	797 (1 study)	⊕⊝⊝⊝ very low^9^
	168 per 1000	98 per 1000 (59 to 155)			
**Signs on bed indicating HIV status** Follow-up: mean 6 months	Study population		OR 0.25 (0.07 to 0.87)	797 (1 study)	⊕⊝⊝⊝ very low^9^
	851 per 1000	587 per 1000 (285 to 832)			
**Marked files indicating HIV status** Follow-up: mean 6 months	Study population		OR 0.54 (0.29 to 1)	797 (1 study)	⊕⊝⊝⊝ very low^9^
	98 per 1000	55 per 1000 (30 to 98)			
	**Control**	**Contact strategy with information giving and empowerment**			
**PLHIV self-esteem** Scale: from 10 to 40 Follow-up: mean one month	The mean PLHIV self-esteem in the control groups was 19.46	The mean PLHIV self-esteem in the intervention groups was 2.12 higher (0.18 to 4.06 higher)		82 (1 study)	⊕⊝⊝⊝ very low^10,11,12^
**PLHIV Workplace stigma** Scale: Not described Follow-up: mean one month	The mean PLHIV workplace stigma in the control groups was 0.46	The mean PLHIV workplace stigma in the intervention groups was 0.31 lower (0.61 to 0.01 lower)		82 (1 study)	⊕⊝⊝⊝ very low^10,11,12^
**Total stigma score** Scale: Not described Follow-up: mean one month	The mean total stigma score in the control groups was 0.42	The mean total stigma score in the intervention groups was 0.17 lower (0.35 lower to 0.01 higher)		82 (1 study)	⊕⊝⊝⊝ very low^10.11.12,13^
**Negative self-perception** Scale: Not described Follow-up: mean one month	The mean self-perception in the control groups was 0.82	The mean self-perception in the intervention groups was 0.46 lower (0.81 to 0.11 lower)		82 (1 study)	⊕⊝⊝⊝ very low^10,11,12^
**Nurses' stigmatizing behaviour** Scale: Not described Follow-up: mean one month	The mean nurses' stigmatizing behaviour in the control groups was 0.46	The mean nurses' stigmatizing behaviour in the intervention groups was 0.07 higher (0.04 lower to 0.18 higher)		86 (1 study)	⊕⊝⊝⊝ very low^10,11,12^
	**Control**	**A 5-day workshop comprising didactic lecture**			
**Empathy** Scale: from 1 to 6 Follow-up: mean 5 days	The mean empathy in the control groups was 4.1	The mean empathy in the intervention groups was 0.2 higher (CI not given, P<0.01		360 (1 study)	⊕⊝⊝⊝ very low^14,15^
**Avoidance attitude** Scale: from 1 to 6 Follow-up: mean 5 days	The mean avoidance attitude in the control groups was 3.5	The mean avoidance attitude in the intervention groups was 0.4 lower (CI not given) P<0.01)		360 (1 study)	⊕⊝⊝⊝ very low^14,15^
**General attitude towards PLHIV** Scale: from -5 to 15 Follow-up: mean 5 day**s**	The mean general attitude towards PLHIV in the control groups was 3.5	The mean general attitude towards PLHIV in the intervention groups was 0.6 higher (CI not given, P<0.01)		360 (1 study)	⊕⊝⊝⊝ very low^14,15^
**Nurses’ willingness to care for PLHIV** Scale: from 0 to 130 Follow-up: mean 5 days	The mean nurses’ willingness to care for PLHIV in the control groups was 97	The mean nurses’ willingness to care for PLHIV in the intervention groups was 13 higher (CI not given, P<0.01)		360 (1 study)	⊕⊝⊝⊝ very low^14,15^
	**Control**	**A one-hour group education (homophobia and fear of death program)**			
**AIDS phobia** Scale from: 15 to 75. Follow up: NA	The mean AIDS phobia in the control groups was 39.49	The mean AIDS phobia in the intervention groups was 0.03 higher (3.13 lower to 3.19 higher		70 (1 study)	⊕⊝⊝⊝ very low^16,17^

^1^ The hospitals were randomized into intervention and control groups. A matched-pair design was applied to optimize the randomization. However, method of the selection of the pairs was not clear. Downgraded one level for risk of bias

^2^ No explanation was given about blinding of allocators

^3^ No control group and the sample sizes at the baseline and post intervention survey are different, hence downgraded two levels for risk of bias

^4^ Wide and statistically non-significant confidence interval

^5^ One control hospital and one experimental hospital was used (conveniently selected), so downgraded one level for risk of bias

^6^ Groups had different scores in fear-based stigma at baseline

^7^ No control group

^8^ The hospitals were conveniently selected. A cross-sectional sample of providers was taken from the selected hospitals. (downgraded one level for risk of bias)

^9^ Cross-sectional nature of data collection, facility characteristics were not considered

^10^ No control group. The intervention sites were conveniently chosen by researchers based on accessibility and willingness to participate. (downgraded one level for risk of bias)

^11^ Five unique case studies were combined, which might have masked differences among the settings

^12^ case series

^13^ Wider confidence interval

^14^ No enough information was given on how lost participants were handled. Around 9% did not provide responses to all questions

^15^ No control group

^16^ No adequate follow up, poor intervention focus.

^17^ Wide confidence interval (additionally downgraded for risk of bias)

**NB:** CI: Confidence Interval, HCWs: Healthcare workers, HIV: Human immunodeficiency virus, PLHIV: People Living with HIV, OR: Odds Ratio, UP: Universal precaution.AIDS: Acquired immunodeficiiency syndrome.

## Discussion

This systematic review attempted to locate, critically appraise, and describe the best available evidence on interventions to reduce HIV-related stigma and discrimination in healthcare settings among HCWs. Studies included in this review employed different measures, intervention types and durations of intervention. Hence, we could not pool the results of the primary studies using meta-analysis.

Previous reviews categorized SAD reduction interventions into the following categories: information-based, structural, biomedical, counseling and support, skills building and contact [17–19]. Most studies included in this review used a combination of two or more interventions to reduce stigma and discrimination in healthcare settings. Information-based approaches used in the studies included in the current review were:

- Training popular opinion leaders through group discussions, games, and role-plays [39].
- Professionally-assisted peer group intervention [62].
- Group education on fear of death and homophobia [67].
- Interactive modular training and discussion focusing on HIV-related stigma, infection control, medical ethics and contact with PLHIV [66].
- Workshops [61, 68], training and dissemination of policy guidelines and educational materials, such as posters on infection control [63, 64].

These information-based approaches were used alone [62, 67] or in combination with others. Among the combinations were: information-based approaches combined with skills building and structural approaches [39]; information-based approaches combined with skills building and contact-based approaches [66]; information-based approaches combined with skills building, structural, contact and biomedical approaches [64]; information-based approaches combined with skills building, structural and contact-based approaches [68]; and information-based with skills building approaches [61].

Although some results reached statistical significance, because of the poor design of the studies, most of the interventions were assigned low or very low quality evidence scores after applying the GRADE approach for assessing methodological quality. Only outcomes reported in one intervention (identifying and training popular opinion leaders, in the presence of adequate supplies) was assigned a moderate quality evidence. This intervention was reported by Li et al. 2015 [39]. The study used both an information-based and structural approach to reduce SAD. The intervention employed diffusion of innovation theory to disseminate information to correct misconceptions related to PLHIV [39]. The intervention was effective in reducing avoidance intent and prejudicial attitudes and in improving compliance to universal precaution. This indicates that structural interventions (availing materials for standard precaution) alone are insufficient, which highlights the necessity of complementing structural interventions with behavioral interventions.

As reported in one study, interventions addressing fear-based stigma reduction through training on basic knowledge of HIV and universal precautions were effective in reducing both social stigma and fear-based stigma [64]. However, the study showed that the effect of the interventions that addressed both fear-based and social stigma was significantly higher in reducing fear-based stigma and extra precaution when compared to interventions addressing fear-based stigma alone [64]. This implies that behavioral interventions targeting stigma and discrimination among healthcare providers should address prejudices and social stigma that may also be part of wider cultural beliefs, in addition to addressing fear-based stigma [69]. Apart from equipping healthcare providers with knowledge and skills, it is paramount to address their emotions [70].

The outcomes reported by all other interventions were assigned very low quality evidence. These interventions included modular interactive training and education on HIV/AIDS [66], professionally-assisted peer group interventions [71], participatory self-guided assessment and interventions,[63] workshops that included didactic lectures [61], and contact-based strategies combined with information provision [68]. All of these interventions resulted in statistically significant reductions in stigma scores. However, a study that used group education on homophobia reported no significant change on AIDS phobia after the intervention [67]. While the improvements observed in most interventions were statistically significant, the design of the studies included in this review were poor. The poor quality of the evidence supporting most of the findings underscores that more rigorous studies such as RCTs are needed to make appropriate decisions for policy and practice.

This review identified five categories of stigma and discrimination reduction interventions: information-based, skills building, structural, contact and biomedical interventions. Unlike other previous reviews [17–19], in this review we did not identify any study conducted among HCWs reporting on the impact of counseling and support approaches to SAD reduction. This may be because, unlike other population groups, HCWs were not provided counseling and support interventions to cope with secondary stigma (stigma that they may face because of their association with PLHIV).

On the other hand, previous studies indicate that HCWs themselves face secondary stigma as a result of their association with PLHIV [31, 32]. Previous studies also showed that HCWs living with HIV face perceived or actual SAD from colleagues or the community [31]. Those studies demonstrated that counseling and support interventions helped to minimize the negative psychosocial impact of HIV-related SAD on clients living with HIV and their families [19, 72–76]. Therefore, the role of counseling and support interventions to reduce internalized or secondary stigma among HCWs should be further investigated.

After we completed this review one meta-analysis and systematic review was published. The study indicated that stigma and discrimination reduction programs resulted in small effect sizes in the improvement of attitudes towards PLHIV [77]. Nevertheless, the subgroup analysis indicated that effect sizes were moderated by the settings in which the intervention was conducted, population type and number of intervention sessions [77]. The review had a limitation in that it was not focused enough with regard to settings, intervention type and population type. Other previous reviews on HIV-related SAD did not provide a focused summary of SAD reduction interventions for specific population groups (such as HCWs) and settings (such as health facilities) or indicate the quality and summary of the findings [17–19]. Although, we could not pool the findings of the studies in the current review because of the heterogeneity of the interventions and outcome measures, we have indicated the quality of evidence for findings reported in this review. These summarized findings may guide policy makers, practitioners and researchers to make appropriate decisions. Nevertheless, the poor quality of evidence supporting most of the findings poses a challenge, especially for practitioners and policy makers.

Most studies were excluded from this review because of poor quality of evidence, mainly related to measurement bias. Therefore, future studies need to fill these gaps. As has been recommended in previous systematic reviews [17, 18], it is important to focus on the design of the studies, which includes paying attention to internal validities and the use of instruments to measure SAD. In addition, there was variability among the measures used in the studies included in the review. This might have been attributed to the absence of standardized measurements. Future studies may fill these gaps as efforts are being made to develop standard tools and instruments to reduce SAD and to monitor these efforts [78]. This, however, will be possible only if the researchers are aware of the recent developments in measurements and scales. Moreover, further study is needed to identify effective interventions to reduce internalized stigma and secondary stigma among HCWs. It is also worth noting that this review did not address health professionals in pre-service training. Hence, knowledge building interventions or curriculum-based stigma reduction interventions have been missed in this review.

## Conclusions

### Implications for practice

Moderate quality evidence indicates that training popular opinion leaders is effective in reducing HCWs’ avoidance intent and prejudicial attitude towards PLHIV and improving compliance to universal precaution. Very low quality evidence indicates that interventions addressing both fear-based stigma and social stigma are more effective in reducing fear-based stigma and the practice extra-precautions when treating HIV positive patients, when compared to interventions addressing only fear-based stigma. Very low quality evidence indicates that the following are effective in reducing stigma-related outcomes: a) professionally-assisted peer group interventions, b) modular interactive training and discussions, c) participatory self-guided assessment and interventions, d) contact strategy with information giving and empowerment, e) workshops comprising didactic lectures. When utilizing the evidence from the current review in policy making and in practice, it is vital to consider the quality of evidence supporting the findings and the limitations of the primary studies reported.

### Implications for research

Further RCTs are needed to provide evidence that guides interventions to reduce HIV-related stigma and discrimination in healthcare settings. Future trials need to use up-to-date instruments to measure stigma and discrimination. Studies are needed to address internalized stigma and secondary stigma among healthcare providers. Further attempts should be made to standardize measures for HIV-related stigma and discrimination.

## Supporting information

S1 Document Critical appraisal instrument for each study design. It indicates critical appraisal instrument for each study design
(DOCX)

S2 Document Studies excluded and reasons for their exclusion. It indicates studies excluded after critical appraisal and reasons for excluding each study.
(DOCX)

S3 Document JBI data extraction instrument. It indicates data extraction tool
(DOCX)

S1 Table PRISMA checklist. It describes the review against the checklist for PRISMA reporting guideline.
(DOCX)

S2 Table Search strategy for all databases. It indicates detailed search strategy for each database.
(DOCX)

## Abbreviations

AIDS: Acquired Immunodeficiency Syndrome
CDC: Centers for Disease Control and Prevention
CI: Confidence interval
DALYs: Disability Adjusted Life Years
GRADE: Grading of Recommendations, Assessment, Development and Evaluation
HCT: HIV Counseling and Testing
HIV: Human Immunodeficiency Virus
JBI: The Joanna Briggs Institute
MD: Mean Difference
NWQ: Nurses’ Willingness Questionnaire
OR: Odds ratio
P value: Probability value
PLC: People Living Close to HIV
PLHIV: People Living with HIV
POL: Popular Opinion Leader
PRISMA: Preferred Reporting Items for Systematic Reviews and Meta-analyses
QOL: Quality of Life
RCT: Randomized Controlled Trial
SD: Standard deviation
SAD: Stigma and discrimination
SOF: Summary of Findings table
UNAIDS: Joint United Nations Programme on HIV/AIDS
USA: United States of America
USAID: United States Aid for International Development

## References

[1] Ortblad KF, Lozano R, Murray CJ. The burden of HIV: insights from the Global Burden of Disease Study 2010. AIDS (London, England). 2013;27(13):2003–17. 10.1097/QAD.0b013e328362ba67 PMC3748855 23660576

[2] Global HIV & AIDS statistics—2018 fact sheet, last accessed on 08/01/2019, available from: http://www.unaids.org/sites/default/files/media_asset/UNAIDS_FactSheet_en.pdf

[3] Joint United Nations Programme on HIV/AIDS. Global AIDS update 2016. Geneva, Switzerland2016.

[4] UNAIDS. UNAIDS Strategy 2016–2021 UNAIDS; 2016 [cited 01/03/2016 01/03/2016]. Available from: http://www.unaids.org/en/aboutunaids/unaidsstrategygoalsby2015.

[5] Pulerwitz J, Bongaarts J. Tackling stigma: fundamental to an AIDS-free future. The Lancet Global Health. 2014;2(6):e311–e2. 10.1016/S2214-109X(14)70219-0 25103291

[6] Feyissa GT, Abebe L, Girma E, Woldie M. Stigma and discrimination against people living with HIV by healthcare providers, Southwest Ethiopia. BMC Public Health. 2012;12:522 10.1186/1471-2458-12-522 22794201 PMC3506482

[7] Nyblade L, Stangl A, Weiss E, Ashburn K. Combating HIV stigma in health care settings: what works? Journal of the International AIDS Society. 2009;12:15–. 10.1186/1758-2652-12-15 19660113 PMC2731724

[8] Stutterheim SE, Sicking L, Brands R, Baas I, Roberts H, Brakel WHv, et al Patient and Provider Perspectives on HIV and HIV-Related Stigma in Dutch Health Care Settings. AIDS PATIENT CARE and STDs. 2014;28(12). 10.1089/apc.2014.0226 PMC4250939 25459231

[9] Grossman CI, Stangl AL. Global action to reduce HIV stigma and discrimination. Journal of the International AIDS Society. 2013;16:18881 10.7448/IAS.16.3.18881 24242269 PMC3833103

[10] Churcher S. Stigma related to HIV and AIDS as a barrier to accessing health care in Thailand: a review of recent literature 2013 1 1, 2013 12–22 p. 10.4103/2224-3151.115829 28612818

[11] Sayles JN, Wong MD, Kinsler JJ, Martins D, Cunningham WE. The association of stigma with self-reported access to medical care and antiretroviral therapy adherence in persons living with HIV/AIDS. Journal of general internal medicine. 2009;24(10):1101–8. 10.1007/s11606-009-1068-8 19653047 PMC2762503

[12] Vanable PA, Carey MP, Blair DC, Littlewood RA. Impact of HIV-related stigma on health behaviors and psychological adjustment among HIV-positive men and women. AIDS and behavior. 2006;10(5):473–82. 10.1007/s10461-006-9099-1 16604295 PMC2566551

[13] Katz IT, Ryu AE, Onuegbu AG, Psaros C, Weiser SD, Bangsberg DR, et al Impact of HIV-related stigma on treatment adherence: systematic review and meta-synthesis. Journal of the International AIDS Society. 2013;16(3 Suppl 2):18640. 24242258 10.7448/IAS.16.3.18640 PMC3833107

[14] Endeshaw M, Walson J, Rawlins S, Dessie A, Alemu S, Andrews N, et al Stigma in Ethiopia: association with depressive symptoms in people with HIV. AIDS Care. 2014;26(8):935–9. 10.1080/09540121.2013.869537 24382290

[15] Logie C, Gadalla TM. Meta-analysis of health and demographic correlates of stigma towards people living with HIV. AIDS Care. 2009;21(6):742–53. 10.1080/09540120802511877 19806490

[16] UNAIDS. HIV-related stigma and human rights violations: case studies of successful programmes 2005 [24/03/2016]. Available from: http://data.unaids.org/publications/irc-pub06/JC999-HumRightsViol_en.pdf.

[17] Stangl AL, Lloyd JK, Brady LM, Holland CE, Baral S. A systematic review of interventions to reduce HIV-related stigma and discrimination from 2002 to 2013: how far have we come? Journal of the International AIDS Society. 2013;16(3 Suppl 2):18734. 24242268 10.7448/IAS.16.3.18734 PMC3833106

[18] Sengupta S, Banks B, Jonas D, Miles MS, Smith GC. HIV interventions to reduce HIV/AIDS stigma: a systematic review. AIDS and behavior. 2011;15(6):1075–87. 10.1007/s10461-010-9847-0 21088989 PMC3128169

[19] Brown L, Macintyre K, Trujillo L. Interventions to reduce HIV/AIDS stigma: what have we learned? AIDS education and prevention. 2003;15(1):49–69. 12627743 10.1521/aeap.15.1.49.23844

[20] Jackson N, Waters E. The challenges of systematically reviewing public health interventions. J Public Health (Oxf). 2004;26(3):303–7. 15454602 10.1093/pubmed/fdh164

[21] Montgomery P, Underhill K, Gardner F, Operario D, Mayo-Wilson E. The Oxford Implementation Index: a new tool for incorporating implementation data into systematic reviews and meta-analyses. Journal of clinical epidemiology. 2013;66(8):874–82. 10.1016/j.jclinepi.2013.03.006 23810026 PMC3746185

[22] Jessica Ogden LN. Common at its Core: HIV-Related Stigma Across Contexts. 2005.

[23] Nyblade L, Hong KT, Anh N, Ogden J, Jain A, Stangl A. Communities confront HIV stigma in Viet Nam: participatory interventions reduce HIV stigma in two provinces. Washington, DC: International Center for Research on Women 2008.

[24] Nyblade L MK. Can we measure HIV/AIDS-related stigma and discrimination? Current knowledge about quantifying stigma in developing countries 2006 [Available from: http://www.policyproject.com/pubs/generalreport/measure%20hiv%20stigma.pdf.

[25] Armstrong R, Waters E, Jackson N. Systematic reviews of health promotion and public health interventions. Melbourne: University of Melbourne 2007.

[26] Moher D, Shamseer L, Clarke M, Ghersi D, Liberati A, Petticrew M, et al Preferred reporting items for systematic review and meta-analysis protocols (PRISMA-P) 2015 statement. Systematic reviews. 2015;4(1):1. 25554246 10.1186/2046-4053-4-1 PMC4320440

[27] Catalin Tufanaru;, Munn Z, Aromataris E, Campbell J, Hopp L. Chapter 3: Systematic reviews of effectiveness In: Aromataris E, Munn Z, editors. Joanna Briggs Institute Reviewer's Manual North Adelaide, South Australia: The Joanna Briggs Institute; 2017.

[28] Shah SM, Heylen E, Srinivasan K, Perumpil S, Ekstrand ML. Reducing HIV Stigma Among Nursing Students: A Brief Intervention. Western journal of nursing research. 2014;36(10):1323–37. 10.1177/0193945914523685 24569699 PMC4459739

[29] Yiu JW, Mak WW, Ho WS, Chui YY. Effectiveness of a knowledge-contact program in improving nursing students' attitudes and emotional competence in serving people living with HIV/AIDS. Social science & medicine (1982). 2010;71(1):38–44. 20430503 10.1016/j.socscimed.2010.02.045

[30] Al-Mazrou YY, Abouzeid MS, Al-Jeffri MH. Impact of health education on knowledge and attitudes of Saudi paramedical students toward HIV/AIDS. Saudi medical journal. 2005;26(11):1788–95. 16311667

[31] Ha PN, Chuc NTK, Hien HT, Larsson M, Pharris A. HIV-related stigma: Impact on healthcare workers in Vietnam. Global public health. 2013;8(sup1):S61–S74. 23738991 10.1080/17441692.2013.799217

[32] Uebel KE, Nash J, Avalos A. Caring for the caregivers: models of HIV/AIDS care and treatment provision for health care workers in Southern Africa. The Journal of infectious diseases. 2007;196(Supplement_3):S500–S4. 18181701 10.1086/521113

[33] USAID. Health Policy Initiative Programmatic Guidance for Reducing HIV and Key Population Stigma and Discrimination: For the Greater Mekong Region Countries of Thailand, Lao PDR and Myanmar. Bangkok, Thailand: USAID; 2012.

[34] Quinn DM, Chaudoir SR. Living with a concealable stigmatized identity: the impact of anticipated stigma, centrality, salience, and cultural stigma on psychological distress and health. Journal of personality and social psychology. 2009;97(4):634 10.1037/a0015815 19785483 PMC4511710

[35] The Joanna Briggs Institute. The Joanna Briggs Institute Critical Appraisal Tools for Use in JBI Systematic Reviews Checklist for Analytical Cross Sectional Studies. North Adelaide, Australia The Joanna Briggs Institute; 2017.

[36] Porritt K, Gomersall J, Lockwood C. JBI's systematic reviews: study selection and critical appraisal. AJN 2014;114(6):47–52. 10.1097/01.NAJ.0000450430.97383.64 24869584

[37] Munn Z, Tufanaru C, Aromataris E. JBI's systematic reviews: data extraction and synthesis. AJN The American Journal of Nursing. 2014;114(7):49–54. 10.1097/01.NAJ.0000451683.66447.89 25742352

[38] Li L, Wu Z, Liang L-J, Lin C, Guan J, Jia M, et al Reducing HIV-related stigma in health care settings: a randomized controlled trial in China. American journal of public health. 2013;103(2):286–92. 10.2105/AJPH.2012.300854 23237175 PMC3556241

[39] Li L, Liang L-J, Lin C, Wu Z. Addressing HIV stigma in protected medical settings. AIDS care. 2015;27(12):1439–42. 10.1080/09540121.2015.1114990 26608559 PMC4689649

[40] GRADEpro G. GRADEpro Guideline Development Tool [Software]. McMaster University 2015.

[41] Bennett C, Weale A. HIV and AIDS awareness: an evaluation of a short training programme for midwives. Journal of advanced nursing. 1997;26(2):273–82. 9292360 10.1046/j.1365-2648.1997.1997026273.x

[42] Baskaran C. Practical Strategies for Educating Nurses in India to Improve Care of Patients with HIV and AIDS. 2014.

[43] Church K, Wringe A, Fakudze P, Kikuvi J, Simelane D, Mayhew SH. Are integrated HIV services less stigmatizing than stand-alone models of care? A comparative case study from Swaziland. Journal of the International AIDS Society. 2013;16:17981 10.7448/IAS.16.1.17981 23336726 PMC3545202

[44] Ezedinachi EN, Ross MW, Meremiku M, Essien EJ, Edem CB, Ekure E, et al The impact of an intervention to change health workers' HIV/AIDS attitudes and knowledge in Nigeria: a controlled trial. Public Health. 2002;116(2):106–12. 10.1038/sj.ph.1900834 11961679

[45] Geibel S, Hossain SMI, Pulerwitz J, Sultana N, Hossain T, Roy S, et al Stigma Reduction Training Improves Healthcare Provider Attitudes Toward, and Experiences of, Young Marginalized People in Bangladesh. Journal of Adolescent Health. 2017;60:S35–S44. 10.1016/j.jadohealth.2016.09.026 28109339

[46] Kaponda CP, Jere DL, Chimango JL, Chimwaza AF, Crittenden KS, Kachingwe SI, et al Impacts of a peer-group intervention on HIV-related knowledge, attitudes, and personal behaviors for urban hospital workers in Malawi. Journal of the Association of Nurses in AIDS Care. 2009;20(3):230–42. 10.1016/j.jana.2008.12.005 19427600 PMC4177099

[47] Neema S, Atuyambe LM, Otolok-Tanga E, Twijukye C, Kambugu A, Thayer L, et al Using a clinic based creativity initiative to reduce HIV related stigma at the Infectious Diseases Institute, Mulago National Referral Hospital, Uganda. African health sciences. 2012;12(2):231–9. 10.4314/ahs.v12i2.24 23056033 PMC3462528

[48] Pisal H, Sutar S, Sastry J, Kapadia-Kundu N, Joshi A, Joshi M, et al Nurses' health education program in India increases HIV knowledge and reduces fear. Journal of the Association of Nurses in AIDS Care. 2007;18(6):32–43. 10.1016/j.jana.2007.06.002 17991597

[49] McKenzie AG. An HIV Educational Tool to Increase Non-HIV Provider Knowledge on the Approach and Management of HIV Patients: Brandman University; 2017.

[50] Pratt R, Pellowe C, Juvekar S, Potdar N, Weston A, Joykutty A, et al Kaleidoscope: a 5‐year action research project to develop nursing confidence in caring for patients with HIV disease in west India. International nursing review. 2001;48(3):164–73. 11558691 10.1046/j.1466-7657.2001.00081.x

[51] Robiner WN, Parker SA, Ohnsorg TJ, Strike B. HIV/AIDS training and continuing education for psychologists. Professional Psychology: Research and Practice. 1993;24(1):35.

[52] Santana RT, Monzon OT, Mandel J, Hall TL, Hearst N. AIDS education for hospital workers in Manila: effects on knowledge, attitudes, and infection control practices. AIDS (London, England). 1992;6(11):1359–64. 1472339

[53] Stewart KE, DiClemente RJ, Ross D. Adolescents and HIV: theory‐based approaches to education of nurses. Journal of advanced nursing. 1999;30(3):687–96. 10499226 10.1046/j.1365-2648.1999.01118.x

[54] Wang D, Operario D, Hong Q, Zhang H, Coates TJ. Intervention to train physicians in rural China on HIV/STI knowledge and risk reduction counseling: preliminary findings. AIDS care. 2009;21(4):468–72. 10.1080/09540120802290357 19266406 PMC2853945

[55] Wu S, Li L, Wu Z, Liang LJ, Cao H, Yan Z, et al A brief HIV stigma reduction intervention for service providers in China. AIDS Patient Care STDS. 2008;22(6):513–20. 10.1089/apc.2007.0198 18462076 PMC2700336

[56] Wu Z, Detels R, Ji G, Xu C, Rou K, Ding H, et al Diffusion of HIV/AIDS knowledge, positive attitudes, and behaviors through training of health professionals in China. AIDS Education and Prevention. 2002;14(5):379–90. 12413184 10.1521/aeap.14.6.379.24074

[57] Li L, Lin C, Guan J, Wu Z. Implementing a stigma reduction intervention in healthcare settings. Journal of the International AIDS Society. 2013;16(3 Suppl 2):18710. 24242261 10.7448/IAS.16.3.18710 PMC3833117

[58] Li L, Guan J, Liang LJ, Lin C, Wu Z. Popular Opinion Leader intervention for HIV stigma reduction in health care settings. AIDS Educ Prev. 2013;25(4):327–35. 10.1521/aeap.2013.25.4.327 23837810 PMC3925348

[59] Li L, Lin C, Guan J. Using standardized patients to evaluate hospital-based intervention outcomes. Int J Epidemiol. 2014;43(3):897–903. 10.1093/ije/dyt249 24369433 PMC4052130

[60] Li L, Liang LJ, Wu Z, Lin C, Guan J. Assessing outcomes of a stigma-reduction intervention with venue-based analysis. Soc Psychiatry Psychiatr Epidemiol. 2014;49(6):991–9. 10.1007/s00127-013-0808-6 24374721 PMC4031272

[61] Williams AB, Wang H, Burgess J, Wu C, Gong Y, Li Y. Effectiveness of an HIV/AIDS educational programme for Chinese nurses. Journal of advanced nursing. 2006;53(6):710–20. 10.1111/j.1365-2648.2006.03777.x 16553679

[62] Norr KF, Norr JL, Kaponda CP, Kachingwe SI, Mbweza EM. Short-term effects of a peer group intervention for HIV prevention among trainee teachers in Malawi. African Journal of AIDS Research. 2007;6(3):239–49. 10.2989/16085900709490420 25866170

[63] Mahendra V, Gilborn L, Bitra G, Samson L, Mudoi R, Jadav S, et al Reducing stigma and discrimination in hospitals: Positive findings from India. Washington, DC: Population Council 2006.

[64] Pulerwitz J, Oanh KT, Akinwolemiwa D, Ashburn K, Nyblade L. Improving hospital-based quality of care by reducing HIV-related stigma: evaluation results from Vietnam. AIDS and behavior. 2015;19(2):246–56. 10.1007/s10461-014-0935-4 25382350

[65] Oanh KTH, Ashburn K, Pulerwitz J, Ogden J, Nyblade L. Improving hospital-based quality of care in Vietnam by reducing HIV-related stigma and discrimination. 2008. 10.1007/s10461-014-0935-4 25382350

[66] Lohiniva AL, Benkirane M, Numair T, Mahdy A, Saleh H, Zahran A, et al HIV stigma intervention in a low-HIV prevalence setting: a pilot study in an Egyptian healthcare facility. AIDS Care. 2016;28(5):644–52. 10.1080/09540121.2015.1124974 26717980

[67] Zachariah GS. AIDS Fear in Health Care Workers: The Development of an Educational Program to Decrease the Fear1998.

[68] Uys L, Chirwa M, Kohi T, Greeff M, Naidoo J, Makoae L, et al Evaluation of a Health Setting-Based Stigma Intervention in Five African Countries. AIDS Patient Care and STDs. 2009;23(12):1059–66. 10.1089/apc.2009.0085 20025515 PMC2832642

[69] Chambers LA, Rueda S, Baker DN, Wilson MG, Deutsch R, Raeifar E, et al Stigma, HIV and health: a qualitative synthesis. BMC Public Health. 2015;15(1):848. 26334626 10.1186/s12889-015-2197-0 PMC4557823

[70] Varas-Díaz N, Neilands TB, Rodríguez-Madera SL, Padilla M. The role of emotions in the reduction of HIV/AIDS stigma among physicians in training. AIDS Care. 2016;28(3):376–83. 10.1080/09540121.2015.1090537 26444133 PMC4747826

[71] Norr KF, Ferrer L, Cianelli R, Crittenden KS, Irarrázabal L, Cabieses B, et al Peer group intervention for HIV prevention among health workers in Chile. Journal of the Association of Nurses in AIDS Care. 2012;23(1):73–86. 10.1016/j.jana.2011.02.001 21497113 PMC3140569

[72] Sikkema KJ, Dennis AC, Watt MH, Choi KW, Yemeke TT, Joska JA. Improving mental health among people living with HIV: a review of intervention trials in low-and middle-income countries. Global Mental Health. 2015;2:e19 10.1017/gmh.2015.17 26435843 PMC4589870

[73] Bateganya M, Amanyeiwe U, Roxo U, Dong M. The Impact of Support Groups for People Living with HIV on Clinical Outcomes: a systematic review of the literature. Journal of acquired immune deficiency syndromes (1999). 2015;68(0 3):S368. 25768876 10.1097/QAI.0000000000000519 PMC4709521

[74] Loutfy M, Tharao W, Logie C, Aden MA, Chambers LA, Wu W, et al Systematic review of stigma reducing interventions for African/Black diasporic women. Journal of the International AIDS Society. 2015;18:19835 10.7448/IAS.18.1.19835 25862565 PMC4393416

[75] Millard T, Elliott J, Girdler S. Self-management education programs for people living with HIV/AIDS: a systematic review. AIDS Patient Care STDS. 2013;27(2):103–13. 10.1089/apc.2012.0294 23298279

[76] Paudel V, Baral KP. Women living with HIV/AIDS (WLHA), battling stigma, discrimination and denial and the role of support groups as a coping strategy: a review of literature. Reproductive health. 2015;12:53 10.1186/s12978-015-0032-9 26032304 PMC4467680

[77] Mak WW, Mo PK, Ma GY, Lam MY. Meta-analysis and systematic review of studies on the effectiveness of HIV stigma reduction programs. Social Science & Medicine. 2017;188:30–40. 28704645 10.1016/j.socscimed.2017.06.045

[78] Carr D, Kidd R., and Nyblade L. Achieving a Stigma-free Health Facility and HIV Services: Resources for Administrators. Washington, DC: Futures Group, Health Policy Project; 2015.

