# Supplementary material for: Reducing HIV-related stigma and discrimination in healthcare settings: A systematic review of quantitative evidence
Source: PLoS One. 2019 Jan 25;14(1):e0211298. doi: 10.1371/journal.pone.0211298 (PMC6347272; doi:10.1371/journal.pone.0211298)
Supplement: S1 Document — It indicates critical appraisal instrument for each study design (DOCX) [file pone.0211298.s001.docx]

**S1 Document: JBI Critical Appraisal instruments**

# JBI critical appraisal checklist for randomized controlled trials

Reviewer Date

Author Year Record Number

|  | Yes | No | Unclear | NA |
| --- | --- | --- | --- | --- |
| 1. Was true randomization used for assignment of participants to treatment groups? | □ | □ | □ | □ |
| 1. Was allocation to treatment groups concealed? | □ | □ | □ | □ |
| 1. Were treatment groups similar at the baseline? | □ | □ | □ | □ |
| 1. Were participants blind to treatment assignment? | □ | □ | □ | □ |
| 1. Were those delivering treatment blind to treatment assignment? | □ | □ | □ | □ |
| 1. Were outcomes assessors blind to treatment assignment? | □ | □ | □ | □ |
| 1. Were treatments groups treated identically other than the intervention of interest? | □ | □ | □ | □ |
| 1. Was follow-up complete, and if not, were strategies to address incomplete follow-up utilized? | □ | □ | □ | □ |
| 1. Were participants analyzed in the groups to which they were randomized? | □ | □ | □ | □ |
| 1. Were outcomes measured in the same way for treatment groups? | □ | □ | □ | □ |
| 1. Were outcomes measured in a reliable way? | □ | □ | □ | □ |
| 1. Was appropriate statistical analysis used? | □ | □ | □ | □ |
| 1. Was the trial design appropriate, and any deviations from the standard RCT design (individual randomization, parallel groups) accounted for in the conduct and analysis of the trial? | □ | □ | □ | □ |

Overall appraisal: Include □ Exclude □ Seek further info □

Comments (Including reason for exclusion)

# JBI critical appraisal checklist for quasi-experimental studies (non-randomized experimental studies)

Reviewer Date

Author Year Record Number

|  | Yes | No | Unclear | Not applicable |
| --- | --- | --- | --- | --- |
| 1. Is it clear in the study what is the ‘cause’ and what is the ‘effect’ (i.e. there is no confusion about which variable comes first)? | □ | □ | □ | □ |
| 1. Were the participants included in any comparisons similar? | □ | □ | □ | □ |
| 1. Were the participants included in any comparisons receiving similar treatment/care, other than the exposure or intervention of interest? | □ | □ | □ | □ |
| 1. Was there a control group? | □ | □ | □ | □ |
| 1. Were there multiple measurements of the outcome both pre-and post the intervention/exposure? | □ | □ | □ | □ |
| 1. Was follow up complete and if not, were differences between groups in terms of their follow up adequately described and analysed? | □ | □ | □ | □ |
| 1. Were the outcomes of participants included in any comparisons measured in the same way? | □ | □ | □ | □ |
| 1. Were outcomes measured in a reliable way? | □ | □ | □ | □ |
| 1. Was appropriate statistical analysis used? | □ | □ | □ | □ |

Overall appraisal: Include □ Exclude □ Seek further info □

Comments (Including reason for exclusion)

# JBI critical appraisal checklist for analytical cross-sectional Studies

Reviewer Date

Author Year Record Number___

|  | Yes | No | Unclear | Not applicable |
| --- | --- | --- | --- | --- |
| 1. Were the criteria for inclusion in the sample clearly defined? | □ | □ | □ | □ |
| 1. Were the study subjects and the setting described in detail? | □ | □ | □ | □ |
| 1. Was the exposure measured in a valid and reliable way? | □ | □ | □ | □ |
| 1. Were objective, standard criteria used for measurement of the condition? | □ | □ | □ | □ |
| 1. Were confounding factors identified? | □ | □ | □ | □ |
| 1. Were strategies to deal with confounding factors stated? | □ | □ | □ | □ |
| 1. Were the outcomes measured in a valid and reliable way? | □ | □ | □ | □ |
| 1. Was appropriate statistical analysis used? | □ | □ | □ | □ |

Overall appraisal: Include □ Exclude □ Seek further info □

Comments (Including reason for exclusion)

# JBI critical appraisal checklist for case Series

Reviewer Date

Author Year Record Number

|  | Yes | No | Unclear | Not applicable |
| --- | --- | --- | --- | --- |
| 1. Were there clear criteria for inclusion in the case series? | □ | □ | □ | □ |
| 1. Was the condition measured in a standard, reliable way for all participants included in the case series? | □ | □ | □ | □ |
| 1. Were valid methods used for identification of the condition for all participants included in the case series? | □ | □ | □ | □ |
| 1. Did the case series have consecutive inclusion of participants? | □ | □ | □ | □ |
| 1. Did the case series have complete inclusion of participants? | □ | □ | □ | □ |
| 1. Was there clear reporting of the demographics of the participants in the study? | □ | □ | □ | □ |
| 1. Was there clear reporting of clinical information of the participants? | □ | □ | □ | □ |
| 1. Were the outcomes or follow up results of cases clearly reported? | □ | □ | □ | □ |
| 1. Was there clear reporting of the presenting site(s)/clinic(s) demographic information? | □ | □ | □ | □ |
| 1. Was statistical analysis appropriate? | □ | □ | □ | □ |

Overall appraisal: Include □ Exclude □ Seek further info □

Comments (Including reason for exclusion)
