# Supplementary material for: Reducing HIV-related stigma and discrimination in healthcare settings: A systematic review of quantitative evidence
Source: PLoS One. 2019 Jan 25;14(1):e0211298. doi: 10.1371/journal.pone.0211298 (PMC6347272; doi:10.1371/journal.pone.0211298)
Supplement: S2 Document — It indicates studies excluded after critical appraisal and reasons for excluding each study. (DOCX) [file pone.0211298.s002.docx]

**S2 Document: Studies excluded and reasons for their exclusion**

| S/n | Study | Reason for exclusion |
| --- | --- | --- |
|  | Bennet 1997[17] | Measurement bias (composite scores not created; scale mean scores were not reported), poor fidelity of the intervention and small sample size |
|  | Baskan,2014[18] | Attitude of nurses toward PLWHAs was not assessed at the pre-test as the AIDS Attitude Scale was introduced only at 12 months follow-up |
|  | Church 2013[19] | Client outcome, instead of HCW outcome, was measured, and the measured outcomes had measurement bias |
|  | Ezedinachi 2002[20] | No measurement scale was created to measure stigma. Single items were used to assess the impact of the intervention. Clear comparison data is not available in the form of mean score and SD. |
|  | Giebel 2017[21] | Did not create composite score or scale to measure stigma |
|  | Kaponda[22] | Differences in HCW characteristics in the baseline and post-intervention, measurement bias (several attitudes measured with only two items, affecting reliability of those measures) stigma items were not developed specifically for health professionals |
|  | Neema 2012[23] | Measurement bias, individual separate items (instead of composite scales) were used to measure attitude. Participants of the pre-test are different from those of post-test. |
|  | McKanzie, 2017[25] | Measurement bias (items were reported separately, no composite score or scale was reported). |
|  | Pisal 2007[24] | Measurement bias (items were reported separately, no composite score or scale was reported). |
|  | Pratt 2001[26] | Did not report outcomes quantitatively. |
|  | Robiner 1994[27] | More intervention groups reported having attended training and more contact with PLHIV than control groups. This poses difficulty in assessing the effect of the intervention (one-day training). The study was planned and implemented only after the intervention (continuing education conference) had already occurred. Pre-test measures were not taken. The intervention’s fidelity was not assured. The size and characteristics (geographically heterogenous) of the sample had limitations. Adequate description was not given on how anxiety was measured |
|  | Santana 1992[28] | Lacks details of measurement scales for attitude |
|  | Stewart 1999[29] | The hypotheses were not aimed to compare attitude, but comfort and intent to perform preventive measures (in performing assessment) the treatment arms. Incomplete data (n1 and n2, SD were not reported) |
|  | Wang 2009[30] | Measurement bias (Physician stigma knowledge was reported) not actual stigma. No scale was described to measure patient stigma |
|  | Wu 2008[31] | Measurement bias (stigma measured and reported separately by three separate items) |
|  | Wu 2002[32] | Measurement bias (empathy was measured by a single item) |
