# Supplementary figures and images for: Reducing HIV-related stigma and discrimination in healthcare settings: A systematic review of quantitative evidence
Source: PLoS One. 2019 Jan 25;14(1):e0211298. doi: 10.1371/journal.pone.0211298 (PMC6347272; doi:10.1371/journal.pone.0211298)

**S3 Document: JBI Data extraction instrument**


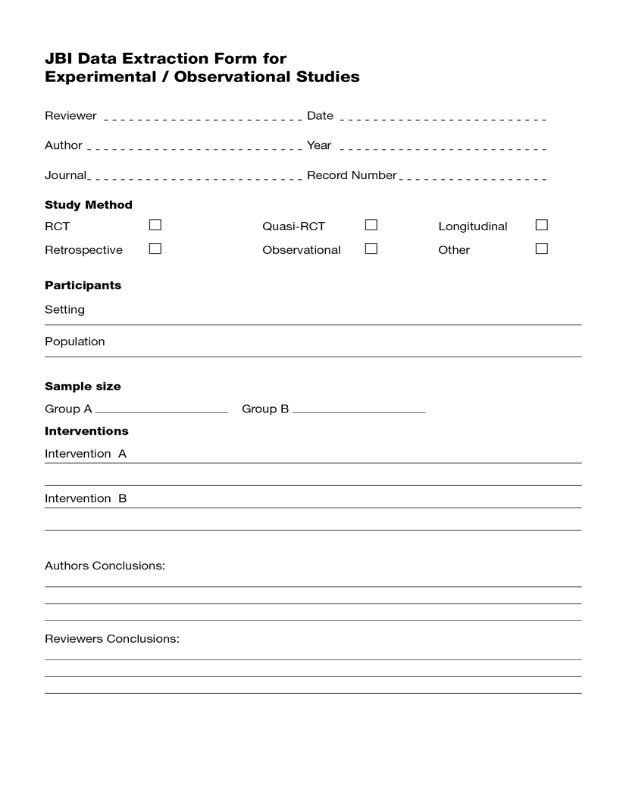


**Insert page break**


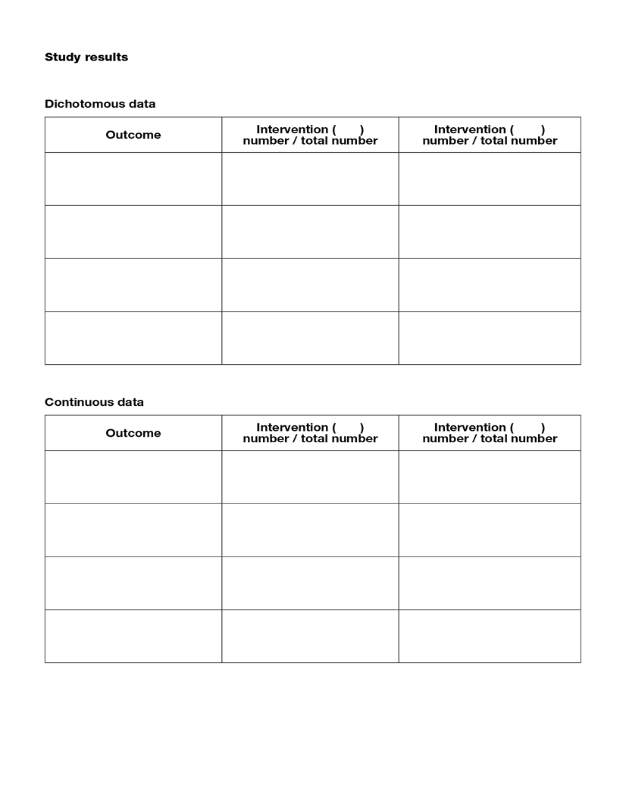

Supplement: S3 Document — It indicates data extraction tool (DOCX) [file pone.0211298.s003.docx]
