# Supplementary material for: Reducing HIV-related stigma and discrimination in healthcare settings: A systematic review of quantitative evidence
Source: PLoS One. 2019 Jan 25;14(1):e0211298. doi: 10.1371/journal.pone.0211298 (PMC6347272; doi:10.1371/journal.pone.0211298)
Supplement: S1 Table — It describes the review against the checklist for PRISMA reporting guideline. (DOCX) [file pone.0211298.s004.docx]

| **Section/topic** | **#** | **Checklist item** | **Reported on page #** |
| --- | --- | --- | --- |
| **TITLE** | | |  |
| Title | 1 | Reducing HIV-related stigma and discrimination in healthcare settings: a systematic review of quantitative evidence (**Page** **1**) |  |
| **ABSTRACT** | | |  |
| Structured summary | 2 | **Introduction**: Stigma and discrimination (SAD) related to HIV compromise access and adherence to treatment and support programs among people living with HIV (PLHIV). The ambitious goal of ending the epidemic of HIV by 2030 set by the United Nations Joint Program of HIV/AIDS (UNAIDS) will thus only be achieved if HIV-related stigma and discrimination are reduced. The objective of this review was to locate, appraise and describe international literature reporting on interventions that addressed HIV-related SAD in healthcare settings.  **Methods**: The databases searched were: Cumulative Index to Nursing and Allied Health (CINAHL), Excerpta Medica Database from Elsevier (EMBASE), PubMed and Psychological Information (PsycINFO) database. Two individuals independently appraised the quality of the papers using appraisal instruments from the Joanna Briggs Institute (JBI). Data were extracted from papers included in the review using the standardized data extraction tool from JBI. Quality of evidence for major outcomes was assessed using Grading of Recommendations, Assessment, Development and Evaluation (GRADE).  **Results**: We retained 14 records reporting on eight studies. Five categories of SAD reduction (information-based, skills building, structural, contact-based and biomedical interventions) were identified. Training popular opinion leaders (POLs) resulted in significantly lower mean avoidance intent scores (MD=-1.87 [95% CI -2.05 to -1.69]), mean prejudicial attitude scores (MD=-3.77 [95% CI -5.4 to -2.09]) and significantly higher scores in mean compliance to universal precaution (MD=1.65 [95% CI 1.41 to 1.89]) when compared to usual care (moderate quality evidence).  **Conclusions**  **Implications for practice:** Evidence of moderate quality indicates that training popular opinion leaders is effective in reducing avoidance intent and prejudicial attitude and improving compliance to universal precaution. Very low quality evidence indicates that professionally assisted peer group interventions, modular interactive training, participatory self-guided assessment and intervention, contact strategy combined with information giving and empowerment are effective in reducing HIV-related stigma.  **Implications for research:** Further Randomized Controlled Trials (RCTs) are needed. Future trials need to use up-to-date and validated instruments to measure stigma and discrimination.(**Page** **2**) |  |
| **INTRODUCTION** | | |  |
| Rationale | 3 | The ambitious goal of ending the epidemic of HIV by 2030 set by the United Nations Joint Program of HIV/AIDS (UNAIDS) will thus only be achieved if HIV-related stigma and discrimination are reduced. Synthesised evidence is needed to design stigma reduction interventions. Through a preliminary search in PubMed, Cumulative Index to Nursing and Allied Health (CINAHL), Excerpta Medica Database from Elsevier (EMBASE) and Cochrane Library, we found no systematic review addressing stigma and discrimination reduction interventions specific to healthcare settings or HCWs published within the last three years. **(Pages** **3-4)** |  |
| Objectives | 4 | This review sought to locate, appraise and describe international literature reporting on interventions that addressed HIV-related stigma and discrimination among healthcare workers and in healthcare institutions.(**Page** **4**) |  |
| **METHODS** | | |  |
| Protocol and registration | 5 | This systematic review was conducted in accordance with an *a-priori* protocol registered in PROSPERO 2017 CRD42017071799 (available from <http://www.crd.york.ac.uk/PROSPERO/display_record.php?ID=CRD42017071799>). (**Page** **5**) |  |
| Eligibility criteria | 6 | This review considered healthcare workers as populations. Interventions that addressed HIV-related SAD by health workers were considered. These included, but were not limited to the following:   1. Information-based approaches including both written and verbal information to increase the understanding of HIV and stigma, provided in the form of leaflets and brochures or through other methods. 2. Skills building approach, such as demonstrations and role-plays. 3. Structural approaches such as availing supplies for standard precautions, revision and development of standard operating procedures, polices and regulations, and putting a system in place for addressing grievances. 4. Contact strategies, such as testimonials of PLHIV and activities that encourage interaction between HCWs and PLHIV. Counseling and support interventions to help cope with HIV-related stigma and discrimination, specifically secondary stigma (stigma that they may face because of their association with PLHIV). 5. Biomedical interventions such as universal access to care and treatment or expansion of HIV counseling and testing (HCT).   The comparators considered were baseline (before intervention), no intervention, usual care and one or more of the above components compared to one another.  The primary outcomes considered for inclusion were HIV-related stigma and discrimination by HCWs in healthcare institutions. Forms of stigma included fear-based stigma, value-based stigma, enacted stigma, internalized stigma or stigma in other forms. Internalized stigma is defined as the acceptance or internalization of shame, blame, hopelessness, guilt, and fear of discrimination associated with being HIV-positive. The secondary outcome considered were PLHIV-specific extra-precaution (excessive use of precautions or over use of protective barriers selectively when handling PLHIV) and adherence to universal precaution.  This review considered all studies conducted worldwide in healthcare settings (hospitals, clinics or health centers).(**Pages** **5**-**7**) |  |
| Information sources | 7 | Both published and unpublished papers reported in the English were searched with no restriction to age of the participants, country and date of publication. The databases searched were: CINAHL, Excerpta Medica Database from Elsevier (EMBASE), PubMed and Psychological information (PsycINFO) database. The search for unpublished studies included: HIVinSite, AIDSinfo, HIV and AIDS clearinghouse, Centers for Disease Control and Prevention (CDC) HIV publications, Health Policy Project (HPP) website, United States Agency for International Development (USAID) experience clearinghouse, and United Nations Joint Program on HIV/AIDS (UNAIDS) publications. (**Page** **7**) |  |
| Search | 8 | A detailed search strategy for each database is reported in supplementary file (**Supplementary** **file** **S1** **Table**). |  |
| Study selection | 9 | Following the above search procedure, all identified citations were loaded into EndNote and duplicates were removed. Titles and abstracts were screened by two independent reviewers for assessment against the inclusion criteria for the review. The full texts of potentially eligible studies were retrieved and assessed in detail against the inclusion criteria by two independent reviewers. (**Page 7)** |  |
| Data collection process | 10 | Quantitative data were extracted from papers included in the review using the standardized data extraction tool from the JBI (S3 doc). Relevant information such as population characteristics, publication year, authors, intervention type and summary of the findings were extracted. Where necessary, primary authors were asked to provide additional information on the articles. Details of data from primary studies with limited data or with limited follow up were checked through request to the authors and through checking subsequent publications from the same project, based on cross-checking the linked publications from the registries of trials (if trial registry number existed). (**Page** **8**) |  |
| Data items | 11 | The primary outcome of the review was stigma. Since there is no single standard measure of stigma, different dimensions and types of stigma were considered for inclusion in the review. Studies that used single or separate items and that did not create composite scales of measurement for attitudinal items of stigma were excluded from the review. (**Pages** **6**-**7**). The primary studies included in this review measured the outcomes as follows:   1. Health care workers’ (HCWs’) avoidance intent was measured using a five-point Likert-scale which assessed HCWs’ willingness to treat PLHIV in eight scenarios. Higher scores indicated a higher intent to avoid service provision to PLHIV. 2. Prejudicial attitude was measured using eight items rated on the five-point Likert scale. 3. Fear-based stigma was measured using twelve items using standardized scales to obtain scores ranging from 1 to 10. For both scales higher scores indicated higher levels of stigma. 4. Public contact stigma was measured using three items on a four-point Likert scale. Since mean item scores (instead of the mean scale score) were reported, possible scores ranged from 1 to 4. 5. Client contact stigma was measured using three items on a three-point Likert scale. Again, mean item scores were reported, so possible scores ranged from 1 to 3. For both scales, higher scores indicated a higher level of stigma. 6. AIDS phobia was measured using 15 items rated on a five-point Likert scale. Hence, the score could range from 15 to 75. (**Pages 14-18)**   The secondary outcome considered were PLHIV-specific extra-precaution (excessive use of precautions or over use of any protective barriers selectively when handling PLHIV) and adherence to universal precautions. We considered both dichotomous measures and continuous scale measures for this outcome.  The included primary studies measured this outcome as follows:   1. Compliance to Universal precaution (UP) was measured using 13 items with responses ranging from 0 (never) to 4 (always) on a Likert scale. Higher scores indicate higher levels of adherence to UP. 2. Utilizing extra precaution (over using protective barriers) (as yes or no) (**Page** **17**) |  |
| Risk of bias in individual studies | 12 | Two individuals (GTF and MS (non-author)) independently appraised the quality of the eligible studies prior to inclusion in the review using appraisal instruments from the Joanna Briggs Institute (JBI) for experimental, quasi-experimental studies and other comparative study designs (S1 doc). After appraisal, studies that did not meet the methodological criteria were excluded and reasons for their exclusion are provided in supplementary file (S2 doc). All disagreements that arose between the reviewers were resolved through discussion, and there was no requirement for a third reviewer. (**Page 8)** |  |
| Summary measures | 13 | Summary measures used in this review were relative risk, odds ratio and mean difference and their confidence intervals. (**Page** **8** and **pages 14-18)** . |  |
| Synthesis of results | 14 | Since the studies were methodologically or clinically heterogeneous, statistical pooling was not possible; hence, the findings are presented in narrative form. (**Page 8)** |  |

Page 1 of 2

| **Section/topic** | **#** | **Checklist item** | **Reported on page #** |
| --- | --- | --- | --- |
| Risk of bias across studies | 15 | The quality of evidence for major outcomes reported was assessed using a software package developed by the Grading of Recommendations, Assessment, Development and Evaluation (GRADE) working group. (**Page** **8**, **Page** **19**) |  |
| Additional analyses | 16 | NA |  |
| **RESULTS** | | |  |
| Study selection | 17 | We have presented the numbers of studies screened, assessed for eligibility, and included in the review in a flow diagram (**Figure** **1**).(**Page** **8**) |  |
| Study characteristics | 18 | We have presented the characteristics of each included study (including their citation details, sample size and characteristics, PICOS, methodological quality, follow-up period and major findings. (**Pages** **9**-**11**) |  |
| Risk of bias within studies | 19 | Risk of bias for all studies has been presented (**Pages 11-13)** |  |
| Results of individual studies | 20 | Though meta-analysis was not practical because of heterogeneity of studies, for each study, we have presented summary data for each intervention group (b) effect estimates and confidence intervals. (**Pages 14-18)** |  |
| Synthesis of results | 21 | NA |  |
| Risk of bias across studies | 22 | The quality of evidence for major outcomes reported was assessed using a software package developed by the Grading of Recommendations, Assessment, Development and Evaluation (GRADE) working group. (**Page 19)** |  |
| Additional analysis | 23 | NA |  |
| **DISCUSSION** | | |  |
| Summary of evidence | 24 | Strength of evidence, implications for policy and practice have been presented (**Pages** **22**-**24**) |  |
| Limitations | 25 | Limitations of the review at study level (risk of bias), at outcome level (presence of only few studies utilizing validated scales, variation in measurements), were discussed. (**Page 24)** |  |
| Conclusions | 26 | Evidence of moderate quality indicates that training popular opinion leaders is effective in reducing avoidance intent and prejudicial attitude and improving compliance to universal precaution. Very low quality evidence indicates that professionally assisted peer group interventions, modular interactive training, participatory self-guided assessment and intervention, contact strategy combined with information giving and empowerment are effective in reducing HIV-related stigma. Further Randomized Controlled Trials (RCTs) are needed. Future trials need to use up-to-date and validated instruments to measure stigma and discrimination.(**Pages 24-25)** |  |
| **FUNDING** | | |  |
| Funding | 27 | The study was conducted as part of GTF’s PhD project which was supported by the Adelaide scholarship International (ASI) granted by the University of Adelaide. The authors did not receive any funding for this research.(**Page 26)** |  |

*From:*  Moher D, Liberati A, Tetzlaff J, Altman DG, The PRISMA Group (2009). Preferred Reporting Items for Systematic Reviews and Meta-Analyses: The PRISMA Statement. PLoS Med 6(7): e1000097. doi:10.1371/journal.pmed1000097

For more information, visit: **www.prisma-statement.org**.

Page 2 of 2
