# Supplementary material for: Reducing HIV-related stigma and discrimination in healthcare settings: A systematic review of quantitative evidence
Source: PLoS One. 2019 Jan 25;14(1):e0211298. doi: 10.1371/journal.pone.0211298 (PMC6347272; doi:10.1371/journal.pone.0211298)
Supplement: S2 Table — It indicates detailed search strategy for each database. (DOCX) [file pone.0211298.s005.docx]

**S2 Table: Search strategy for specific databases**

**CINAHL**

Last search date =23/07/2017

| **S/n** | **Query** | **Result** |
| --- | --- | --- |
| **1** | (MH "HIV-Infected Patients+") OR (MH "HIV-AIDS Nursing") OR (MH "HIV Infections+") OR (MH "HIV Education") OR (MH "HIV-1") OR "HIV" OR (MH "AIDS Serodiagnosis") | 70307 |
| **2** | (MH "AIDS Serodiagnosis") OR (MH "AIDS Patients") OR (MH "Attitude to AIDS") OR (MH "HIV-AIDS Nursing”) | 8597 |
| **3** | (MH "Acquired Immunodeficiency Syndrome") OR (MH "Human Immunodeficiency Virus+”) | 15866 |
| **4** | (MH "Discrimination") OR (MH "Prejudice") OR (MH "Stigma") OR (MH "Stereotyping") OR (MH "Homophobia") OR "prejudices" OR "Stigma" OR “prejudice” | 23174 |
| **5** | MM "Psychological Well-Being" OR (MH "Adaptation, Psychological") | 20672 |
| **6** | 1 OR 2 OR 3 | 71029 |
| **7** | 4 OR 5 | 43308 |
| **8** | 6 AND 7 | 4363 |
| **9** | Limit 8 by [English Language] AND [Exclude MEDLINE AND limit to humans | 777 |

**Cochrane**

Last search date: 20/05/17

| **S/n** | **Query** | **Result** |
| --- | --- | --- |
| 1 | MeSH descriptor: [HIV] explode all trees | 2943 |
| 2 | MeSH descriptor: [Acquired Immunodeficiency Syndrome] explode all trees | 1267 |
| 3 | MeSH descriptor: [Social Stigma] explode all trees | 113 |
| 4 | MeSH descriptor: [Adaptation, Psychological] explode all trees | 4750 |
| 5 | MeSH descriptor: [Resilience, Psychological] explode all trees | 123 |
| 6 | MeSH descriptor: [Stereotyping] explode all trees | 335 |
| 7 | MeSH descriptor: [Self Concept] explode all trees | 5760 |
| 8 | MeSH descriptor: [Quality of Life] explode all trees | 19694 |
| 9 | #1 or #2 | 3935 |
| 10 | #3 or #4 or #5 or #6 or #7 or #8 | 28673 |
| 11 | #9 and #10 in Trials | 142 |

**EMBASE**

Last search date: 18/06/2017

| **S/n** |  |  |
| --- | --- | --- |
| **1** | ‘human immunodeficiency syndrome virus’/exp OR ‘acquired immune deficiency syndrome’/exp OR ‘human immune deficiency syndrome’: ab,ti | 135, 583 |
| **2** | ‘acquired immunodeficiency syndrome’: ab,ti OR ‘human immunodeficiency virus’:ab,ti OR ‘human immune deficiency virus’ OR ‘acquired immune-deficiency syndrome’:ab,ti OR ‘acquired immune-deficiency syndrome’:ab,ti OR ‘human immune-deficiency virus’:ab,ti | 95,815 |
| **3** | ‘social stigma’/exp OR ‘social discrimination’/exp OR ‘stereotype’/exp | 29,168 |
| **4** | ‘prejudice’/exp OR ‘prejudice’:ab,ti | 5032 |
| **5** | 1 OR 2 | 205,768 |
| **6** | 3 OR 4 | 33,762 |
| **7** | 5 AND 6 | 1004 |
| **8** | #7 AND [EMBASE]/lim NOT [Medline]/lim | 301 |
| **9** | #8 AND ('clinical article'/de OR 'clinical trial'/de OR 'comparative study'/de OR 'controlled study'/de OR 'evidence based medicine'/de OR 'evidence based practice'/de OR 'human'/de OR 'pilot study'/de OR 'randomized controlled trial'/de) | 274 |
|  | #9 AND ('clinical article'/de OR 'clinical trial'/de OR 'comparative study'/de OR 'controlled study'/de OR 'evidence based medicine'/de OR 'evidence based practice'/de OR 'human'/de OR 'pilot study'/de OR 'randomized controlled trial'/de) AND [humans]/lim AND [English]/lim | 266 |

**PsycINFO**

Last search date: 20/05/2017

| **S/n** | **Query** | **Result** |
| --- | --- | --- |
| 1 | Exp HIV/ | 38093 |
| 2 | (Human immune deficiency syndrome OR acquired immune deficiency syndrome).sh,ti,ab | 2877 |
| 3 | exp "AIDS (ATTITUDES TOWARD)"/ OR exp AIDS/ | 15258 |
| 4 | exp Social Stigma/ or exp Stereotyping/ or exp Prejudice/ or exp discrimination/ or exp Violence/ or exp Domestic Violence/ or exp Workplace Violence/ | 133991 |
| 5 | exp Social Discrimination/ or exp Stigma/ or exp "AIDS (Attitudes Toward)"/ or exp "Physical Illness (Attitudes Toward)"/ | 21977 |
| 6 | (Stigma* OR discrimination OR prejudice* OR labeling OR stereotyp* OR disclosure).sh,ti,ab | 152385 |
| 7 | Exp Quality of Life/ OR exp Social Support/ OR exp Coping Behavior/ OR exp Emotional Adjustment/ | 121878 |
| 8 | (coping OR cope OR self-management OR bereave* OR (quality of life)).ti,ab | 144748 |
| 9 | judgment/ OR Stereotyped Attitudes/ OR Blame/ OR exp guilt/ OR shame/ or embarrassment/ | 40426 |
| 10 | 1 OR 2 OR 3 | 38756 |
| 11 | 4 OR 5 OR 6 OR 7 OR 8 OR 9 | 448676 |
| 12 | 10 AND 11 | 10101 |
| 13 | Limit 12 to (human and english language) | 9737 |
| 14 | Limit 13 to (“0300 clinical trial” OR “0410 experimental replication” OR “1900 scientific simulation” OR “2100 treatment outcome”) | 216 |

**ProQuest Dissertations and Theses**

Last updated search on 10/06/2017

| **S/n** | **Query** | **Result** |
| --- | --- | --- |
| 1 | ti(hiv 1) OR ti(hiv 2) OR ti(hiv) OR ti(acquired immunodeficiency syndrome) OR ti(human immunodeficiency virus)= | 9776 |
| 2 | su(hiv 1) OR su(hiv 2) OR su(hiv) OR su(acquired immunodeficiency syndrome) OR su(human immunodeficiency virus) | 7383 |
| 3 | su(stigma hiv) OR diskw(Stigma) OR ti(stigma) OR su(hiv stigma) OR ti(discrimination) OR su(prejudice) OR ti(prejudice) OR su(aids attitude) | 8626 |
| 4 | 1 OR 2 | 11,326 |
| 5 | 3 AND 4 | 283 |
| 6 | Limit #5 to English Language | 279 |

**PubMed**

Last search date: 20/05/2017

| S/n | Query | Results |
| --- | --- | --- |
| 1 | "Acquired Immunodeficiency Syndrome"[Mesh] OR "HIV"[Mesh] OR "HIV Infections"[Mesh] | 281060 |
| 2 | “acquired immune deficiency syndrome”[tw] OR “acquired immunodeficiency syndrome” [tw] OR “Human immunodeficiency virus” [tw] OR “Human immune deficiency virus” [tw] OR “acquired immuno-deficiency syndrome” [tw] OR “acquired immune-deficiency syndrome” [tw] OR “human immune-deficiency virus” [tw] OR “human immune-deficiency virus” [tw] OR HIV[tw] OR hiv-1*[tw] OR hiv-2*[tw] OR hiv1[tw] OR hiv2[tw] OR HIV infect*[tw] OR HIV/AIDS[tw | 358968 |
| 3 | ((“Social Stigma” [Mesh]) OR (“Discrimination (Psychology)”[Mesh] OR “Social Discrimination” [Mesh])) OR “Prejudice”[Mesh] | 50181 |
| 4 | Stigma* [tiab] OR discrimination [tiab] OR prejudice [tiab] | 120425 |
| 5 | Disclosure [MESH] OR Truth Disclosure [Mesh] OR Self Disclosure [Mesh] | 35243 |
| 6 | "Resilience, Psychological"[Mesh] OR "Adaptation, Psychological"[Mesh] OR "Emotional Adjustment"[Mesh] OR "Stress, Psychological"[Mesh] OR "Self-efficacy"[Mesh] OR "Self-concept"[Mesh] OR "Self-psychology"[Mesh] OR "Self-Care"[Mesh] | 325427 |
| 7 | 1 OR 2 | 363703 |
| 8 | 3 OR 4 OR 5 OR 6 | 492714 |
| 9 | 7 AND 8 | 15878 |
| 10 | Limit #9 to (Comparative Study[ptyp] OR Evaluation Studies[ptyp] OR Pragmatic Clinical Trial[ptyp] OR Randomized Controlled Trial[ptyp] OR Clinical Trial[ptyp]) | 1253 |
| 11 | Limit #10 AND humans AND English AND AIDS | 1215 |
